# Engineering the Dynamics of Cell Adhesion Cues in Supramolecular Hydrogels for Facile Control over Cell Encapsulation and Behavior

**DOI:** 10.1002/adma.202008111

**Published:** 2021-08-01

**Authors:** Mani Diba, Sergio Spaans, Simone I. S. Hendrikse, Maartje M. C. Bastings, Maaike J. G. Schotman, Johnick F. van Sprang, Dan Jing Wu, Freek J. M. Hoeben, Henk M. Janssen, Patricia Y. W. Dankers

**Affiliations:** ^1^ Institute for Complex Molecular Systems Eindhoven University of Technology P.O. Box 513 Eindhoven MB 5600 The Netherlands; ^2^ Laboratory of Chemical Biology Department of Biomedical Engineering Eindhoven University of Technology P.O. Box 513 Eindhoven MB 5600 The Netherlands; ^3^ Laboratory for Cell and Tissue Engineering Department of Biomedical Engineering Eindhoven University of Technology P.O. Box 513 Eindhoven MB 5600 The Netherlands; ^4^ SyMO‐Chem B.V. Den Dolech 2 Eindhoven AZ 5612 The Netherlands

**Keywords:** cell encapsulation, dynamic hydrogels, molecular exchange dynamics, supramolecular biomaterials, synthetic extracellular matrix

## Abstract

The extracellular matrix (ECM) forms through hierarchical assembly of small and larger polymeric molecules into a transient, hydrogel‐like fibrous network that provides mechanical support and biochemical cues to cells. Synthetic, fibrous supramolecular networks formed via non‐covalent assembly of various molecules are therefore potential candidates as synthetic mimics of the natural ECM, provided that functionalization with biochemical cues is effective. Here, combinations of slow and fast exchanging molecules that self‐assemble into supramolecular fibers are employed to form transient hydrogel networks with tunable dynamic behavior. Obtained results prove that modulating the ratio between these molecules dictates the extent of dynamic behavior of the hydrogels at both the molecular and the network level, which is proposed to enable effective incorporation of cell‐adhesive functionalities in these materials. Excitingly, the dynamic nature of the supramolecular components in this system can be conveniently employed to formulate multicomponent supramolecular hydrogels for easy culturing and encapsulation of single cells, spheroids, and organoids. Importantly, these findings highlight the significance of molecular design and exchange dynamics for the application of supramolecular hydrogels as synthetic ECM mimics.

## Graphical abstract

Slow‐ and fast‐exchanging molecules are co‐assembled into transient fibrous networks, forming supramolecular hydrogels. Modulating the ratio between these molecules can dictate the extent of dynamic behavior of the hydrogels and incorporated bioactive cues. The dynamic character of supramolecular building blocks and the tunability of this system are exploited for facile encapsulation of cellular components and control over their behavior.

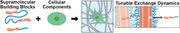

## 1 Introduction

Natural extracellular matrix (ECM) in most tissues is a hydrogel‐like fibrous network that hierarchically forms based on directed interactions between small and large molecules, which dictate their assembly into transient supramolecular fibers.^[^ ^1^ ^]^ This dynamic matrix exhibits various biophysical and biochemical cues that regulate cellular behavior, and therefore plays a central role in vital processes such as tissue growth and regeneration.^[^ ^2^, ^3^ ^]^ Accordingly, substantial research effort has been devoted toward the development of synthetic hydrogels that can serve as ECM mimics for 3D culture of cells and organoids.^[^ ^4^, ^5^ ^]^ Such synthetic materials must be biocompatible, and should ideally incorporate bioactive cues that instruct cell behavior.^[^ ^6^, ^7^, ^8^, ^9^, ^10^ ^]^

Recently, dynamic hydrogels based on non‐covalent or dynamic covalent chemistry are increasingly preferred as synthetic ECM mimics.^[^ ^11^, ^12^ ^]^ Supramolecular hydrogels are dynamic hydrogels that assemble from molecular building blocks through directed, non‐covalent interactions.^[^ ^13^, ^14^ ^]^ The reversibility of such non‐covalent interactions renders these materials adaptable.^[^ ^12^ ^]^ This adaptability allows for matrix remodeling and cellular activities such as cell spreading and migration to take place within the hydrogel matrix, without requiring hydrogel degradation or large pore sizes that are otherwise essential in conventional synthetic hydrogels.^[^ ^12^, ^15^, ^16^ ^]^ Various types of natural and synthetic polymers, such as hyaluronic acid^[^ ^17^ ^]^ and poly(ethylene glycol) (PEG),^[^ ^18^, ^19^ ^]^ have been modified with complementary or self‐complementary supramolecular motifs. These motifs form inter‐ and intra‐molecular cross‐links via interactions such as host–guest or hydrogen bonding, which can create 3D interconnected gel networks at sufficiently high cross‐linking densities.^[^ ^20^ ^]^

The assembly processes, the morphologies of assembled structures, and overall properties of supramolecular materials depend not only on the type of supramolecular interactions at play, but also on the molecular design of the covalent framework of the supramolecular building blocks.^[^ ^21^, ^22^, ^23^ ^]^ For supramolecular hydrogels formed from molecules with bivalent (B) or monovalent (M) fourfold hydrogen bonding designs, we previously observed that viscoelastic properties heavily depend on the ratio between these building blocks.^[^ ^24^ ^]^ Recently, we found that the exchange dynamics of these M‐ and B‐type molecules within and between assembled supramolecular fibers can be tuned by varying their ratio.^[^ ^25^ ^]^

In dynamic hydrogels formed by ionic or host–guest cross‐links, recent findings highlight the key role of biophysical cues, such as stiffness and stress relaxation, on cellular activity and fate.^[^ ^26^, ^27^, ^28^ ^]^ However, contradictory outcomes have been observed upon functionalization of dynamic hydrogels with biochemical cues. While the incorporation of integrin‐binding arginine–glycine–aspartate (RGD) ligands is found to enhance cell spreading in some dynamic hydrogel systems,^[^ ^26^ ^]^ other systems have been shown not to benefit from such functionalization strategies.^[^ ^27^ ^]^ As of yet, key factors determining the effective functionalization of dynamic hydrogels with biochemical cues are not well understood.

**Figure 1:**
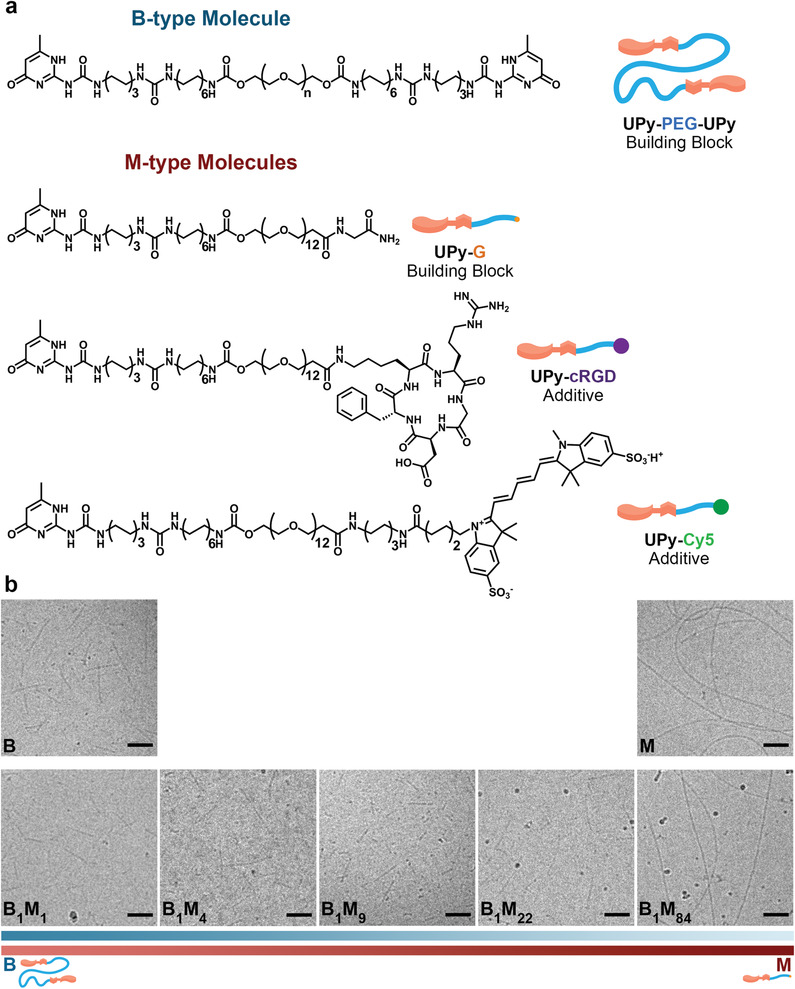
Supramolecular building blocks and their self‐assembly into fibers. a) Molecular structures of bivalent (B)‐type and monovalent (M)‐type molecules as the supramolecular building blocks and additives. For UPy‐PEG‐UPy, *n* is on average 226 (*M*_n_ = 10 kDa). b) Representative Cryo‐TEM images showing the morphology of fibers assembled from B‐type and M‐type molecules at different molar ratios. The ureido‐pyrimidinone (UPy)‐based molecules self‐assemble into fibers (of multiple stacks) at physiological pH and temperature, through dimerization via quadruple hydrogen bonds, and lateral stacking induced by flanking urea moieties forming a hydrophobic pocket shielded from the water. Scale bars = 100 nm.

Here, we investigate the effect of molecular exchange dynamics on incorporation of bioactive motifs in supramolecular hydrogels. To this end, we use B‐ and M‐type molecules containing ureido‐pyrimidinone (UPy) groups as supramolecular building blocks. UPy groups dimerize via fourfold hydrogen bonding with a binding constant and a life‐time of 6 × 10^7^ M^−1^ and ≈1 s in chloroform, respectively.^[^ ^29^ ^]^ In water, however, these hydrogen bonds need to be shielded. In our design, this is achieved by incorporation of an alkyl spacer, forming a hydrophobic pocket upon assembly of UPy‐dimers into 1D stacks, which are further stabilized by hydrogen bonding of flanking urea groups (**Figure** **1**).^[^ ^30^ ^]^ Further assembly occurs by bundling of the stacks into fibers.^[^ ^19^ ^]^ By altering the ratio between B‐ and M‐type monomers in this system, we modulate the molecular exchange dynamics in the assembled fibers and therefore the resulting hydrogels. Subsequently, using rheology, fluorescence photo‐bleaching, and cell studies, the exchange dynamics are identified as an important factor determining the effectiveness of functionalization with cell‐binding motifs. Consequently, the non‐gel‐forming regimes of B‐ and M‐type UPy‐based molecules are used to formulate a multicomponent supramolecular hydrogel system as synthetic ECM for 3D encapsulation and culture of cells, spheroids, and organoids.

## 2 Results and Discussion

Two types of supramolecular building blocks were designed for the formulation of our supramolecular hydrogels (Figure 1a). As the B‐type building block (i.e., UPy‐PEG‐UPy), a PEG chain with a molecular weight of 10 kDa was end‐capped with two UPy moieties. As the M‐type building block (i.e., UPy‐G), an oligo(ethylene glycol) (OEG) chain with a molecular weight of 528 Da was end‐capped with a UPy moiety at one end and a glycine‐amide group at the other end. The glycine‐amide group was included in this design as a biomimetic alternative to the methoxy end groups employed in previously reported M‐type molecules.^[^ ^24^, ^25^ ^]^ In this case, the peripheral glycine‐amide groups are expected to be presented to cells, while the OEG is shielded from exposure, thereby potentially minimizing the biological consequences of the glycols’ non‐fouling properties. Finally, two variants of M‐type molecules were synthesized containing a sulfonated cyanine dye (UPy‐Cy5) or a cyclic RGD (UPy‐cRGD) end group, as fluorescent or cell‐adhesive supramolecular additives, respectively.

The building blocks (with or without additives) were first dissolved at predefined concentrations and ratios in aqueous solutions using an alkaline pH at which the unbound UPy groups were deprotonated.^[^ ^19^ ^]^ The fiber formation was then induced by neutralizing the solutions. Hereafter, we denote the molar ratios of B and M components for each composition as B*_X_* M*_Y_*, where *X* and *Y* indicate the relative molar content of B‐ and M‐type molecules, respectively. Cryogenic transmission electron microscopy (Cryo‐TEM) showed that B‐type molecules assemble into relatively short fibers with a fiber length of ≈160 ± 100 nm, and M‐type molecules into significantly longer fibers of microscale length (Figure 1b and Figure S1, Supporting Information). The addition of a low amount of B‐type molecules to M‐type molecules (B_1_M_84_) did not alter the length of the resulting fiber. However, higher B contents (e.g., B_1_M_22_) impeded the long fiber assembly, most likely due to the high dynamicity of B‐type molecules disrupting the lateral stacking of M‐type dimers.^[^ ^24^, ^25^ ^]^ We propose that one of the factors determining the higher dynamicity of B‐type molecules in this system originates from the significantly longer and polydisperse nature of the ethylene glycol chain (≈19 times longer), as compared to the shorter, monodisperse chain present in the M‐type molecules. Consequently, these long hydrophilic entities in B‐type molecules destabilize the packing of the assembled cores of the fibers due to entropic and steric mechanisms,^[^ ^25^, ^31^ ^]^ yielding fibers with relatively higher molecular exchange dynamics. In contrast, the shorter and monodisperse ethylene glycol chain in the M‐type molecules results in less hydrophilic molecules, favoring intermolecular interactions and therefore “tighter” packing into less dynamic structures, which is reflected by the calculated partition coefficients of 0.29 and −4.73 for M‐type (i.e., UPy‐G) and B‐type molecules, respectively.

The Cryo‐TEM images also showed an average diameter within a range of 8–10 ± 2 nm for the fibers of the studied compositions. The fiber contrast in these images originates only from the hard block, consisting of UPy and alkyl parts of the molecules, as ethylene glycol chains display no contrast relative to the aqueous background.^[^ ^32^ ^]^ Accordingly, these results suggest that fiber formation in this system involves the clustering of ≈3–4 discrete stacks per individual fiber.

**Figure 2:**
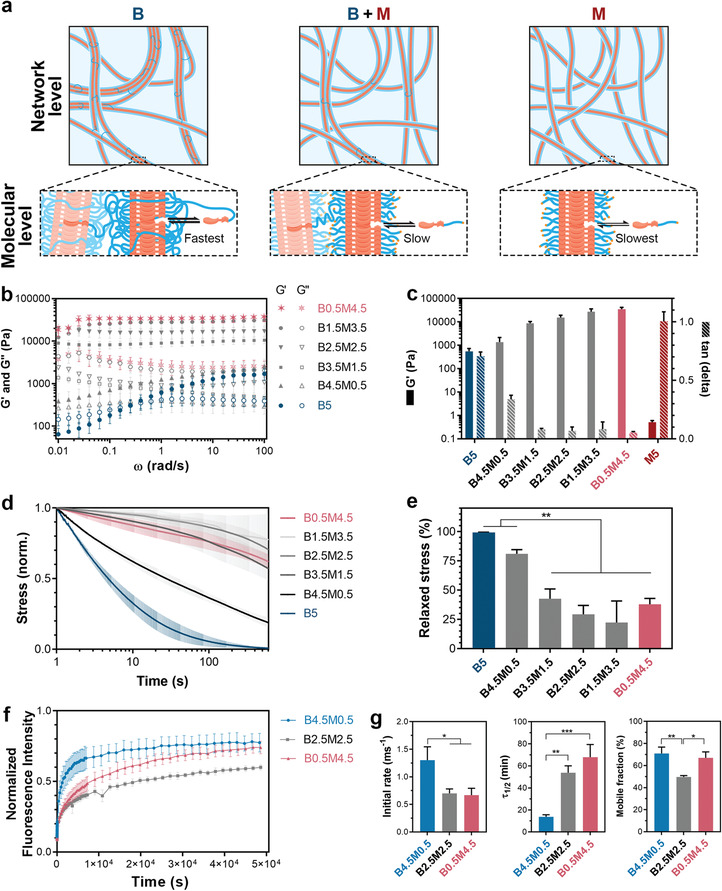
Hydrogels formulated from different ratios of supramolecular building blocks. a) Schematic illustration of different supramolecular formulations at network and molecular levels. Blue linkages between fibers at network level indicate interfiber cross‐links formed by B‐type molecules, and labels of black arrows at molecular level indicate the rate of molecular exchange dynamics for different formulations. b) Frequency dependence of storage (*G*′) and loss (*G*″) moduli of different compositions of supramolecular hydrogels. c) *G*′ and damping factor (tan(delta)) values of hydrogels measured at 1 rad s^−1^ and 1% strain. d) Stress relaxation behavior of supramolecular hydrogels measured by subjecting the hydrogels to 1% strain. e) Quantification of stress relaxation in hydrogels after 10 min. f) Fluorescence recovery after photo‐bleaching (FRAP) tests performed on hydrogels containing 20 µm of UPy‐Cy5 supramolecular additives. g) Quantified FRAP results showing the rate of fluorescence recovery during the first 60 s after photo‐bleaching (Initial rate), the timespan during which the Cy5 fluorescence intensity recovers to half its mobile fraction (τ_1/2_), and the fraction of fluorescence intensity that recovers when fluorescence intensity curves reach plateau values (Mobile fraction). b–g) All hydrogels contained a total polymer content of 5 wt%, and all measurements were performed at 37 °C. All data are shown for *n* = 3 independent tests per group, and as mean ± s.d. e,g) *, *p* < 0.05; **, *p* < 0.01; ***, *p* ≤ 0.001; one‐way analysis of variance (ANOVA) followed by Bonferroni post hoc.

Increasing the concentration of building blocks in solution can result in the formation of a hydrogel network owing to the entanglement and bundling of assembled fibers (**Figure** **2**a and Figure S2, Supporting Information). We altered the ratio between the building blocks at a fixed total concentration of 5 wt%, to study the effect of co‐assembly. Hereafter, we denote the samples as B*Z*M*W*, where *Z* and *W* indicate the wt% of B‐ and M‐type molecules in each composition, respectively (see Table S1, Supporting Information, for an overview of hydrogel compositions).

While at 5 wt% the B‐type building blocks (UPy‐PEG‐UPy) formed hydrogels, the M‐type building blocks (UPy‐G) solely were incapable of forming hydrogels, as they failed the inverted‐vial test (Figure S3, Supporting Information) and exhibited tan(delta) ≥ 1 in rheological measurements (Figure S4, Supporting Information). Nevertheless, co‐assembly of M‐ and B‐type molecules even with low contents of B‐type molecules (e.g., B0.5M4.5, with a molar ratio of B_1_M_84_) resulted in the formation of hydrogels, indicating that B‐type molecules can intercalate into M‐type fibers and act as effective interfiber cross‐linkers in this system due to the B nature of these molecules. This co‐assembly approach was highly effective and resulted in solid‐like behavior (i.e., *G*′ > *G*″) at building block concentrations as low as 0.02 wt% for a fixed molar ratio of B_1_M_84_ (Figure S5, Supporting Information). Nonetheless, our rheological studies showed that hydrogels formed at lower M/B ratios exhibited a more frequency‐dependent viscoelastic response, were less stiff (lower storage modulus [*G*′] values), and showed a less solid‐like behavior(higher tan(delta) values) (Figure 2b,c). Similar behavior was previously observed for M‐type molecules with a methoxy end‐group,^[^ ^24^ ^]^ and can be attributed to the higher dynamicity of the B‐type molecules disrupting the vitrification of less dynamic M‐type stacks, as well as the shorter length of such fibers observed in Figure 1b.

Next, the effect of M/B ratio on the dynamic behavior of the gel networks was studied with stress relaxation experiments. To this end, a strain of 1% was applied and the decay of the generated stress in the hydrogel networks was monitored over a period of 10 min (Figure 2d). Hydrogels formed entirely from B‐type molecules (B5) displayed complete stress relaxation during the course of the experiment, while exhibiting a relaxation half‐life (τ_1/2_) of ≈5 s (Figure 2d) . Notably, increasing the M‐type relative to the B‐type content resulted in hydrogels with slower stress relaxation, which can be attributed to a reduced dynamic behavior of the gel network due to slower molecular rearrangement. At these hybrid compositions (e.g., B0.5M4.5), while the available B‐type molecules establish interfiber cross‐links required for stable network formation, the high content of M‐type molecules renders “tightly packed” fibers that can entangle and exhibit a higher resistance against stress relaxation. Accordingly, these experiments revealed that the network dynamics and stress relaxation in the hydrogels could be varied by altering the M/B ratio.

To elucidate whether the dynamic properties of the gel networks were also altered at the molecular level, we performed fluorescence recovery after photo‐bleaching (FRAP) experiments by including traceable M‐type fluorescent additives, UPy‐Cy5, in the hydrogel compositions (Figure 2f). These UPy‐Cy5 molecules closely resemble the design of UPy‐G molecules and were aimed to co‐assemble with the UPy‐based building blocks, as reported previously.^[^ ^25^, ^33^, ^34^ ^]^ The FRAP experiments revealed that the dynamic properties of the hydrogels were also dampened at the molecular level at higher M/B ratios, as the M‐type additives showed slower recovery kinetics in B0.5M4.5 (τ_1/2_ = 4073 ± 689 s) and B2.5M2.5 (τ_1/2_ = 3227 ± 372 s) as compared to the B4.5M0.5 (τ_1/2_ = 823 ± 115 s) hydrogels (Figure 2f,g). Nonetheless, it should be noted that the higher affinity of the UPy‐Cy5 molecules to the compositions with a higher M/B ratio can be also impacted by the higher concentration of UPy moieties present in these samples (Table S1, Supporting Information). Interestingly, despite the similarities between the recovery kinetics (initial rate and τ_1/2_) of B0.5M4.5 and B2.5M2.5 groups, the B2.5M2.5 hydrogels exhibited a significantly lower fraction of mobile molecules (50 ± 1%) as compared to the B0.5M4.5 group (67 ± 5%), indicating fundamental differences between the exchange behavior of UPy‐based additives in these groups. This larger fraction of immobile molecules in B2.5M2.5 suggests existence of complex subdiffusion phenomena in this composition involving combinations of fast and slow exchange processes.^[^ ^35^ ^]^ Elucidating the differences in molecular exchange dynamics of B2.5M2.5 and B0.5M4.5 compositions in the hydrogel state experimentally remains a challenge. However, our Förster resonance energy transfer (FRET) measurements have shown distinct differences in FRET signal when comparing the pristine dispersions of M‐ or B‐type fibers at a low, non‐gel‐forming, building block concentration of 24.75 µm (Figure S6, Supporting Information). Such FRET measurements can potentially enable future investigations of differences among the molecular exchange dynamics of co‐assembled samples at shorter time scales. Importantly, our previous FRET experiments have shown that the molecular exchange dynamics of comparable M‐type molecules enormously change upon co‐assembly with comparable B‐type molecules, resulting in increased exchange dynamics owing to disordering of the M‐type molecule packing.^[^ ^25^ ^]^

Notably, the FRAP tests performed on hydrogels containing UPy‐free Cy5 molecules indicated the lack of co‐assembly of these additive molecules with the building blocks, as the Cy5 molecules could freely diffuse within the hydrogel matrix preventing their detectable photo‐bleaching within the timeframe of the experiment (data not shown).

**Figure 3:**
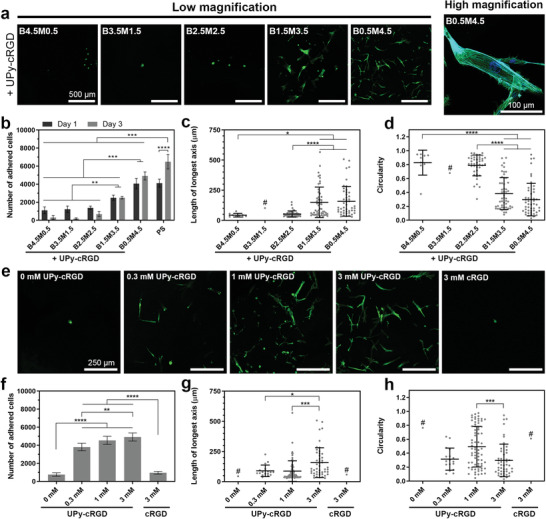
Cell adhesion and spreading on hydrogels with different compositions. a) Representative images of HVSCs after 1 day of culture on different supramolecular hydrogel compositions. b) Number of cells adhered onto hydrogel surfaces after 1 and 3 days of culture. PS indicates polystyrene control. c) Length of longest axis and d) circularity of cells after 1 day of culture on supramolecular hydrogels with different compositions. a–d) Hydrogels contained 3 mm of UPy‐cRGD additives. e) Representative images of HVSCs after 1 day of culture and f) number, g) length of longest axis, h) and circularity of cells adhered after 1 (g,h) or 3 (f) days of culture on B0.5M4.5 supramolecular hydrogels containing different concentrations of UPy‐cRGD or cRGD additives. a,e) Green and blue colors in images indicate actin and nucleus staining, respectively. b–d,f–h) *, *p* < 0.05; **, *p* < 0.01; ***, *p* ≤ 0.001; ****, *p* ≤ 0.0001; two‐way (b) or one‐way (c,d,f–h) analysis of variance (ANOVA) followed by Bonferroni post hoc. All results were obtained from three to four biologically independent experiments per group, and all values are shown as mean ± s.d. # indicates the groups for which the number of cells present was insufficient for statistically relevant comparison. c,d,g,h) Data points represent features of individual cells, with *n* comprising the total number of cells per group that were detectable/analyzed among three experiments.

We hypothesized that the identified differences in dynamic behavior of the hydrogels formulated at different M/B ratios would determine the exchange dynamics of bioactive additives in the hydrogels and their effects on cells. To evaluate this hypothesis, we studied the adhesion and spreading of cells in contact with different hydrogel compositions, as fundamental read‐outs indicative of cell–matrix interactions. Specifically, cell adhesion and spreading are principal requirements for directing function of a wide range of cells types, and can shed light on the potential applicability of our findings in different biomedical arenas.^[^ ^36^, ^37^ ^]^ To this end, 3 mm of M‐type adhesive ligands, UPy‐cRGD, were included in hydrogels of different compositions, and the adhesion and morphology of vascular‐derived matrix‐producing myofibroblasts (human vena saphena cells; HVSC) cultured on hydrogel surfaces were studied. Upon UPy‐cRGD inclusion, the viscoelastic properties (*G*′, *G*″ and stress relaxation behavior) of the hydrogels remained similar (Figure S7, Supporting Information). A high cell viability (>90%) was observed for all compositions upon 3 days of culture, with no statistically significant difference among different hydrogels, suggesting cytocompatibility of this supramolecular system (Figure S8, Supporting Information). Nonetheless, our results revealed that the UPy‐cRGD molecules were ineffective (cell circularity > 0.6 at Day 1; <300 cells at Day 3) in hydrogels with a high content of B‐type molecules, whereas cell adhesion and spreading increased significantly with an increase in the M/B ratio of hydrogels (**Figure** **3**a–d). Cell adhesion and spreading were particularly remarkable for cells seeded on hydrogels of B0.5M4.5 composition (cell circularity = 0.3 ± 0.2 at Day 1; 4917 ± 448 cells at Day 3). Furthermore, the intracellular bundles of F‐actin filaments (i.e., stress fibers) were especially prominent in this group, emphasizing superior cell–matrix adhesion through the formation of focal adhesions.^[^ ^38^, ^39^ ^]^ These results indicate that excessively dynamic hydrogels are not rendered cell‐adhesive upon incorporation of UPy‐cRGD molecules, highlighting the importance of molecular exchange dynamics for the effective incorporation of adhesive ligands in the hydrogels. It is proposed that the lack of cell adhesiveness arises from the low molecular stability of the transient fiber structures, not providing a sufficient retention of incorporated bioactive motifs as required for the formation of focal adhesions by cells via integrin‐cRGD binding.

To understand the fate of M‐type additives in the hydrogels, we studied the release of UPy‐Cy5 molecules from different hydrogel compositions upon their immersion in PBS solutions. Notably, all the hydrogel compositions displayed a similar UPy‐Cy5 release profile (≈20% cumulative release at Day 3 for all 5 wt% compositions; Figure S9, Supporting Information), confirming that the difference in cellular response to the hydrogels is due to differences in their dynamic behavior at the molecular level and is not caused by the release of additives into solutions from the non‐cell‐adhesive compositions. Nonetheless, the hydrogels with a lower M/B ratio showed enhanced erosion (e.g., 38.3 ± 0.7% for B4.5M0.5 after 7 days), which can be attributed to the degradation and release of their B‐type building blocks.

To confirm that the obtained results are applicable to different cell types, cardiomyocyte progenitor cells (CMPCs) were also cultured on the hydrogels of different compositions, which followed a similar behavior as observed for HVSCs, indicating that these results are not limited to an individual cell type (Figure S10, Supporting Information). As B0.5M4.5 composition displayed optimal cell attachment and spreading, we chose this M/B ratio for further investigations.

Next, we investigated the effect of UPy‐cRGD concentration in the hydrogels on cell adhesion and spreading. Without UPy‐cRGD additives, cell adhesion was minimal and cells exhibited spherical morphology at the hydrogel surface, indicating that cRGD adhesive ligands were responsible for cell adhesion onto the hydrogels (Figure 3e–h). Upon inclusion of 0.3 mm of UPy‐cRGD in the hydrogels, cell adhesion onto the hydrogels increased significantly and the cells displayed spread‐out morphology, which continued to improve by increasing the UPy‐cRGD concentration. However, non‐modified cRGD additives (i.e., without UPy moiety) were ineffective to promote cell attachment since these molecules do not incorporate in the M‐type fiber and therefore are prone to burst release, as observed for non‐modified Cy5 additives (65.3 ± 1.4% cumulative release at Day 3 from B0.5M4.5 hydrogels; Figure S9, Supporting Information).

It is worth pointing out that the observation of inferior cell adhesion onto hydrogels with excessively dynamic behavior (Figure 3b) or without UPy‐cRGD additives (Figure 3f) was followed with additional detachment of weakly adhered cells during the processing (washing) steps necessary for cell staining/imaging, resulting in a low number of cells detectable for microscopic analysis (e.g., one remaining cell detected for 0 mm UPy‐cRGD hydrogels in Figure 3g). Therefore, we have not included these groups in statistical analyses and have drawn the conclusions above based solely on the quantification of the number of adhered cells (Figure 3b,f) and the morphological features (Figure 3c,d,g,h) in the other groups with sufficient numbers of detectable cells.

**Figure 4:**
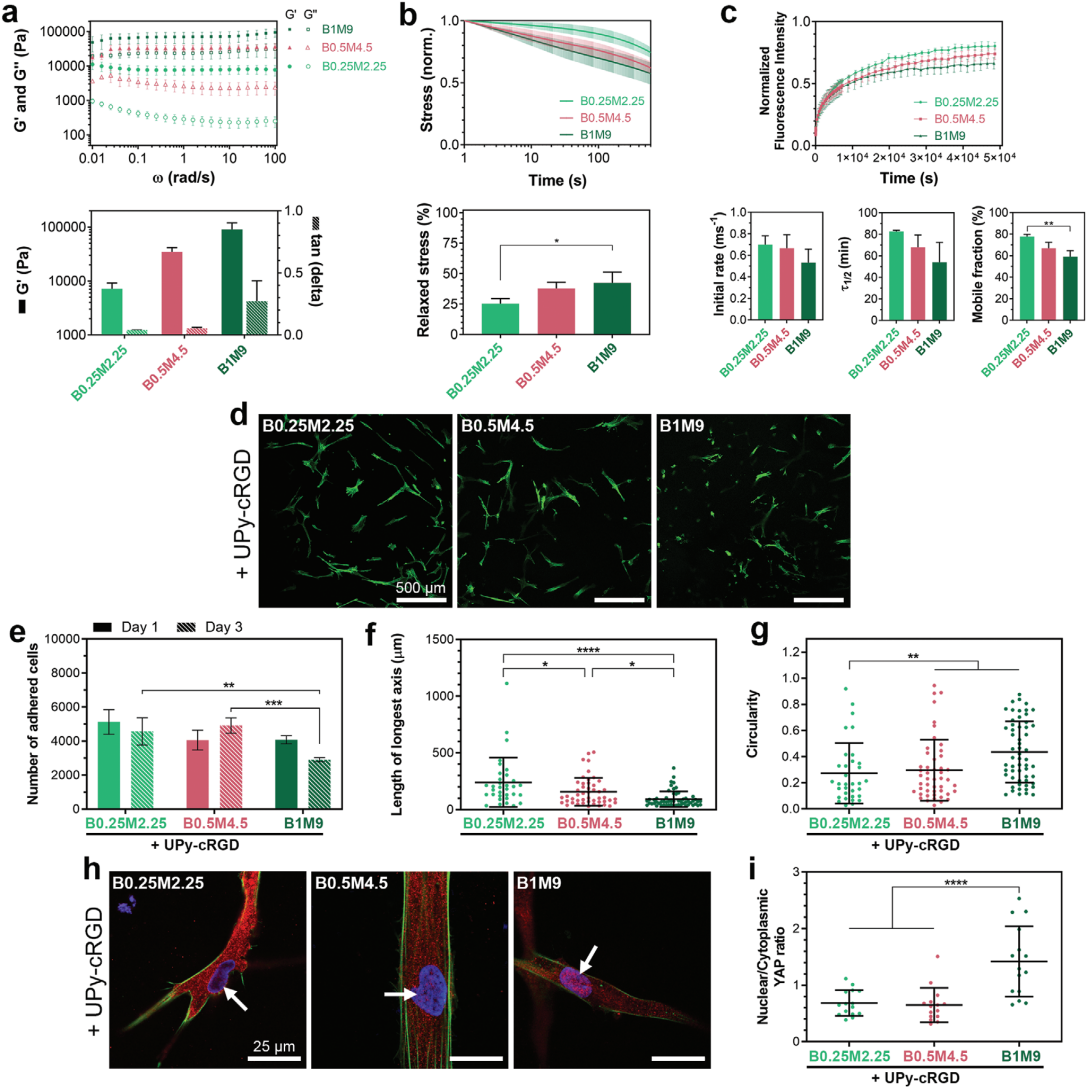
Concentration‐dependent behavior of hydrogels. a) Frequency dependence of viscoelastic behavior, and quantified storage moduli (*G*′) and tan(delta) values (at 1 rad s^−1^ and 1% strain) of hydrogels with different total polymer concentrations and a fixed M/B ratio. b) Stress relaxation behavior of supramolecular hydrogels measured by subjecting the hydrogels to 1% strain. c) Fluorescence recovery after photo‐bleaching (FRAP) tests performed on hydrogels containing 20 µm of UPy‐Cy5 additives. Quantified results show the rate of fluorescence recovery during the first 60 s after photo‐bleaching (Initial rate), the timespan during which the fluorescence intensity recovers to half its mobile fraction (τ_1/2_), and the fraction of fluorescence intensity that recovers when fluorescence intensity curves reach plateau values (Mobile fraction). a–c) All measurements were performed at 37 °C. d) Representative images of HVSCs after 1 day of culture on hydrogels with different polymer concentrations. Green color in images indicates actin staining. e) Number of cells adhered onto hydrogel surfaces after 1 and 3 days of culture. f) Length of longest axis and g) circularity of cells after 1 day of culture on supramolecular hydrogels. h) Representative images of HVSCs upon immunofluorescence staining for nucleus (blue), actin (green), and YAP (red) after 1 day of culture on hydrogels with different polymer concentrations. Arrows indicate the nuclei. i) Quantification of the nuclear/cytoplasmic ratio of the YAP concentration in cells after 1 day of culture. d–i) Hydrogels contained 3 mm of UPy‐cRGD additives. b,c,e,f,g,i) *, *p* < 0.05; **, *p* < 0.01; ***, *p* ≤ 0.001; ****, *p* ≤ 0.0001; one‐way (b,c,f,g,i) or two‐way (e) analysis of variance (ANOVA) followed by Bonferroni post hoc. All results were obtained from three to four independent experiments per group, and all values are shown as mean ± s.d. f,g,i) Data points represent features of individual cells, with *n* comprising the total number of cells per group that were detectable/analyzed among three experiments.

We then investigated the effect of total polymer content on the hydrogels properties by altering the polymer concentration within a range of 2.5–10 wt% at a fixed M/B ratio. Changing the polymer concentration altered the storage modulus of the hydrogels (**Figure** **4**a), whereas the network and molecular dynamics of the hydrogels were not largely affected (Figure 4b,c). All hydrogels displayed a high degree of cell attachment and spreading (Figure 4d–g). A slight decrease in cell number and spreading was observed upon increasing polymer concentration to 10 wt% (i.e., B1M9), which might be due to the higher PEG content inherent to this concentration. Highly hydrophilic PEG chains are known to exhibit anti‐fouling properties,^[^ ^40^ ^]^ and have been previously exploited to render supramolecular biomaterials non‐cell‐adhesive.^[^ ^41^, ^42^ ^]^ Therefore, the long PEG chains might possibly influence cell behavior via potential shielding of the bioactive RGD cell‐adhesive ligands in the supramolecular hydrogels. Consequently, this necessitates clarifying the possible role of PEG content in the differences observed in cell adhesion and spreading on the hydrogels with different compositions (Figure 3 and Figure S11a, Supporting Information). Ideally, designing B‐ and M‐type molecules with PEG spacers of similar length would allow for complete exclusion of possible role of PEG content from the system. We, therefore, synthesized B‐ and M‐type molecules with shorter and longer PEG spacers (5 kDa), respectively, as compared to the current design. Our experimental attempts, however, revealed that these alternatively designed molecules are not suitable for hydrogel formation, as the B‐type molecules with a shorter PEG chain were insoluble in water, while M‐type molecules with a longer PEG spacer produced hydrogels with excessively rapid solubility (complete dissolution at 37 °C in less than 24 h). Nonetheless, more in‐depth analyses of the above‐discussed results can partially rule out the PEG content as the driving factor determining the cellular behavior in this system. First, a direct comparison of the total PEG content of different hydrogel compositions (Figure S11a, Supporting Information) clarifies that B1M9 contained a higher PEG content (5.1 wt%) than all non‐adhesive compositions of B4.5M0.5 (4.3 wt% PEG content), B3.5M1 (3.9 wt% PEG content), and B2.5M2.5 (3.4 wt% PEG content). Despite this higher PEG content, the B1M9 hydrogels successfully facilitated cell adhesion and spreading, while these other compositions (with UPy‐cRGD additives) failed at this role. Second, if the anti‐fouling properties of longer PEG chains present in the B‐types molecules are the driving force behind the lack of cell adhesion in supramolecular hydrogels, a higher molar ratio between the UPy‐cRGD additives and B‐type molecules in hydrogel compositions would result in enhanced cell adhesion and spreading. However, while the cell‐adhesive B0.5M4.5 composition with 0.3 mm UPy‐cRGD content exhibited a UPy‐cRGD/B molar ratio of 0.67, all hydrogel compositions of B2.5M2.5, B3.5M1.5, and B4.5M0.5 with 3 mm UPy‐cRGD content exhibited a higher UPy‐cRGD/B molar ratio (Figure S11b, Supporting Information) yet were not able to support cell adhesion and spreading (Figure 3). Nonetheless, these findings cannot fully rule out the potential effect of long PEG chains on cell adhesion at the molecular level. One potential phenomenon could be the formation of a shield‐like layer on the surface of fibrous assemblies for compositions with a high relative content of B‐type molecules. Such an anti‐fouling layer might essentially block the access of cell integrin receptors to the UPy‐cRGD additives embedded within the fibers, thereby shielding these cell binding motifs for a range of hydrogel compositions. Due to the above‐discussed experimental limitations of the current UPy‐based system, this potential phenomenon might be investigated in future studies by systematic comparison with other supramolecular hydrogel systems that are proposed to show higher molecular exchange dynamics, such as those based on benzene‐1,3,5‐tricarboxamide motifs.^[^ ^14^ ^]^

On a separate note, our results indicate that differences in cell response to different compositions of hydrogels did not originate from altered elasticity, as B0.25M2.25 and B3.5M1.5 exhibited statistically identical storage moduli (*G*_B0.25M2.25_ = 7.2±2.0 kPa and *G*″_B3.5M1.5_ = 8.8±1.6 kPa; *p* = 0.33), but cell behavior on their surface was significantly different.

To analyze the behavior of cells in contact with the supramolecular hydrogels beyond the above‐discussed adhesion and morphological features, we further studied the nuclear localization of Yes‐associated protein (YAP) in cells cultured on different hydrogels. YAP is widely known as a transcriptional regulator that acts as a universal mechanotransducer, mediating the cellular response to the mechanical cues of the ECM.^[^ ^43^, ^44^ ^]^ Consequently, the YAP nuclear‐cytoplasmic translocation has been correlated with cellular changes in response to materials with different stiffness, degradability, or stress relaxation.^[^ ^26^, ^43^, ^45^ ^]^ Our results indicated that altering the M/B ratio (B1.5M3.5 vs B0.5.M4.5) and UPy‐cRGD concentration (1 mm vs 3 mm) among the cell‐adhesive compositions did not affect the YAP translocation in cells cultured onto these hydrogels (Figure S12, Supporting Information). Nonetheless, when comparing hydrogels with different total polymer concentrations, we observed a significant nuclear localization of YAP for cells cultured on the B1M9 group (Figure 4h,i). This significant YAP translocation can be attributed to the highly elastic nature of this composition (*G*′ ≈ 90 kPa), as observed previously for 2D culture of cells on such stiff substrates.^[^ ^43^ ^]^

**Figure 5:**
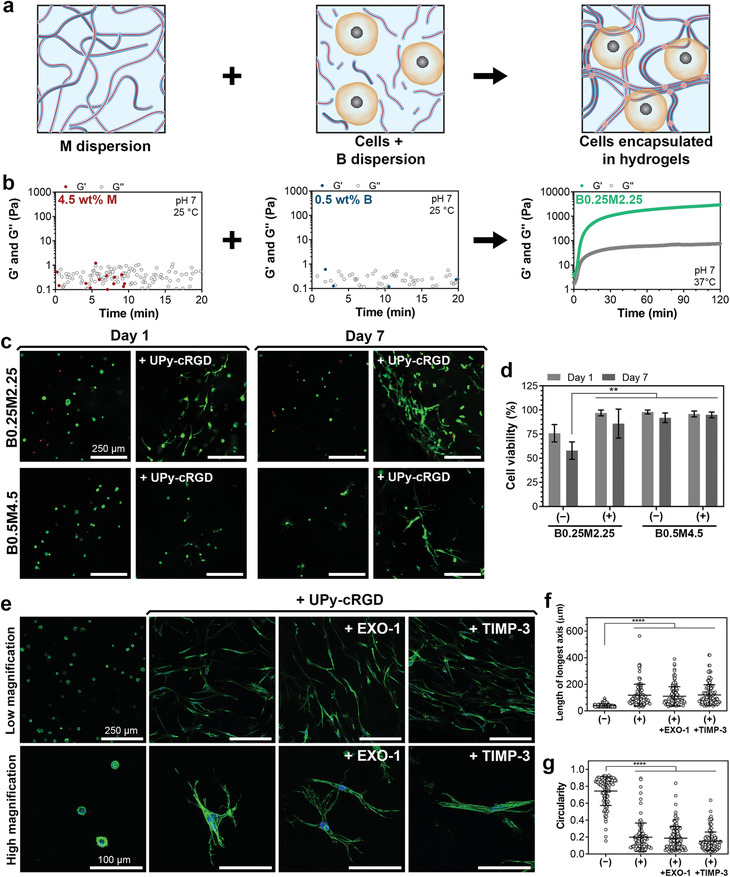
Cell encapsulation and spreading in supramolecular hydrogels. a) Schematic illustration of cell encapsulation in hydrogels via mixing of pre‐assembled supramolecular fibers. b) Representative viscoelastic properties of dispersions of 4.5 wt% M or 0.5 wt% B supramolecular fibers and their mixture measured over time. c) Representative images of HVSCs encapsulated within supramolecular hydrogels without or with 3 mm of UPy‐cRGD additives, after live (green color) and dead (red color) staining. d) Quantification of viability of cells encapsulated in the hydrogels without (−) or with (+) 3 mm of UPy‐cRGD additives, as shown in (c). e) Representative images of HVSCs encapsulated in supramolecular hydrogels of B0.25M2.25 composition without or with 3 mm of UPy‐cRGD additives after 3 days of culture. During the culture period, additional EXO‐1 (120 nm) or TIMP‐3 (5 nm) treatments are carried out to block exocytosis and protein remodeling, respectively. Green and blue colors in images indicate actin and nucleus staining, respectively. f) Length of longest axis and g) circularity of cells after 3 days of culture in supramolecular hydrogels without (−) or with (+) 3 mm of UPy‐cRGD additives, as shown in (e). d) **, *p* < 0.01; two‐way analysis of variance (ANOVA) followed by Bonferroni post hoc. f,g) ****, *p* ≤ 0.0001; one‐way ANOVA followed by Bonferroni post hoc. All biological results were obtained from three independent experiments per group, and their values are shown as mean ± s.d. f,g) Data points represent features of individual cells, with *n* comprising the total number of cells per group that were detectable/analyzed among three experiments.

Nonetheles, 3D encapsulation of cells within the hydrogel matrix is indeed a requirement for the successful application of these materials as synthetic ECM. Notably, we observed that M‐type UPy‐G molecules were incapable of forming hydrogels by themselves, and B‐type UPy‐PEG‐UPy molecules did not gelate at low concentrations of <1 wt% (**Figure** **5**a,b). This unique combination of features enabled the possibility to exploit the non‐gel‐forming regimes of both B‐ and M‐type molecules in this system to develop a mixing‐induced gelation method for cell encapsulation within the hydrogels at physiological pH and temperature. In this strategy, cells were included in a dispersion of B‐type fibers, and gel formation was initiated upon mixing two separate dispersions, one composed of M‐type, and the other composed of B‐type molecules (Figure 5a,b). The co‐formulation process resulted in stable hydrogels within 15 min after mixing the two components. Following this approach, HVSCs were encapsulated in 2.5 wt% (B0.25M2.25) and 5 wt% (B0.5M4.5) hydrogels, with or without UPy‐cRGD additives. It should be noted that this mixing‐induced gelation method is mainly applicable to compositions with a high M/B ratio, as higher concentrations of B‐type molecules can self‐gelate at physiological pH,^[^ ^46^ ^]^ preventing the necessary mixing of B‐ and M‐type components in the dispersion state.

Nearly all cells remained viable after 1 and 7 days of culture in all hydrogels, except for 2.5 wt% hydrogels without UPy‐cRGD in which cells showed reduced viability (58 ± 9%) upon 7 days of culture (Figure 5c,d). The reduced viability in this experimental group can be attributed to the absence of sufficient matrix interactions resulting in anoikis,^[^ ^47^ ^]^ which has been commonly observed for hydrogels free of adhesive ligands.^[^ ^48^, ^49^, ^50^ ^]^ Cell spreading was evident in both 2.5 and 5 wt% hydrogels that contained UPy‐cRGD (Figure 5c and Figure S13, Supporting Information). In the 5 wt% hydrogels, however, cell spreading appeared to be delayed as compared to 2.5 wt% samples, possibly as consequence of the higher elasticity of the 5 wt% hydrogels.

To determine whether these two hydrogel concentrations exhibit significantly different pore sizes, water‐soluble fluorescent FITC‐Dextran macromolecules with average molecular weights of 20 kDa (Stokes radius ≈ 3 nm), 100 kDa (Stokes radius ≈ 7 nm), or 2000 kDa (Stokes radius ≈ 27 nm) were incorporated within the hydrogels, and their diffusion was studied through FRAP measurements. Smaller macromolecules (≤100 kDa) could freely diffuse within the pores of the hydrogels and could not be photo‐bleached due to their high diffusivity, whereas larger macromolecules (2000 kDa) were photo‐bleached and displayed similar recovery profiles in both hydrogel concentrations (for B0.25M2.25 and B0.5M4.5, τ_1/2_ was 6.9 ± 0.1 and 5.8 ± 0.2 s, respectively; Figure S14, Supporting Information). These results indicate the presence of similar submicron pore sizes within gel networks at both concentrations of 2.5 and 5 wt%.

To elucidate the mechanism of cell spreading in the supramolecular hydrogels developed in the current study, we investigated cell spreading in response to the inhibition of nascent protein deposition and remodeling. A recent study highlighted that early protein deposition can significantly impact the behavior of cells encapsulated within dynamic hydrogel matrices.^[^ ^27^ ^]^ The authors did not observe enhanced spreading for mesenchymal stromal cells upon functionalization of a dynamic hyaluronic acid hydrogel with RGD ligands, and concluded that the mechanism responsible for cell spreading in dynamic hydrogels is not mainly driven by tethered adhesive ligands.

To determine whether this previously proposed mechanism is valid in our system, EXO‐1 (2‐(4‐fluorobenzoylamino)‐benzoic acid methyl ester) or TIMP‐3 (tissue inhibitor of metalloproteinase 3) were used to block exocytosis and protein remodeling within hydrogels, respectively. Upon 3 days of culture, HVSCs encapsulated in B0.25M2.25 hydrogels with UPy‐cRGD additives displayed spread‐out morphology (Figure 5e). Remarkably, in contrast to the results observed previously for dynamic hyaluronic acid hydrogels,^[^ ^27^ ^]^ EXO‐1 or TIMP‐3 addition did not suppress the spreading of the cells in these hydrogels. These results signify that for supramolecular hydrogels with a tuned dynamic profile, adhesive ligands can overrule the possible effects of deposition and remodeling of nascent proteins on early cell spreading.

**Figure 6:**
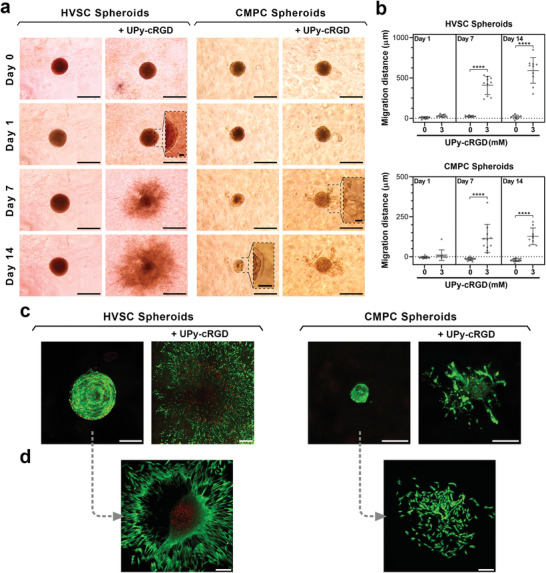
Multicellular spheroids encapsulated in supramolecular hydrogels. a) Representative images of HVSC and CMPC spheroids encapsulated in supramolecular hydrogels without or with 3 mm of UPy‐cRGD additives. Scale bars = 500 µm (main images of HVSC spheroids), 250 µm (main images of CMPC spheroids), and 50 µm (insets). b) Quantification of migration distance of cells from the initial surface of spheroids into hydrogel matrices. ****, *p* ≤ 0.0001; one‐way analysis of variance (ANOVA) followed by Bonferroni post hoc. Results were obtained from four biologically independent experiments per group, and the values are shown as mean ± s.d. Data points represent cell migration distance from initial spheroid surface, with *n* comprising the total number of spheroids per group that were detectable/analyzed among four experiments. c) Representative images of HVSC and CMPC spheroids after 14 days of culture in supramolecular hydrogels without or with 3 mm of UPy‐cRGD additives. d) Representative images of HVSC and CMPC cells after 2 days of culture of spheroids extracted from hydrogels without UPy‐cRGD. c,d) Green and red colors indicate live and dead cells, respectively; Scale bars = 200 µm. a–d) All hydrogels were of B0.25M2.25 composition.

Spheroids and organoids are multicellular 3D structures that exhibit more biological resemblance to natural tissues compared to single cells, and are therefore increasingly used for regenerative medicine and as tools to study diseases.^[^ ^51^ ^]^ Although cell–cell interactions play a major role in these 3D cellular constructs, cell–matrix interactions can also highly impact their behavior such as growth and differentiation.^[^ ^52^ ^]^ Thus, we investigated the potential of our supramolecular hydrogels to direct the behavior of multicellular spheroids. We hypothesized that effective incorporation of adhesive ligands into supramolecular hydrogels can direct spheroids’ behavior via altering cell–matrix interactions at the hydrogel‐spheroid interface. Additionally, these experiments were intended to determine whether the hydrogel compositions with reduced dynamic nature (e.g., B0.25M2.25) still exhibited sufficient matrix adaptability to allow for cellular activities such as cell migration in 3D space. To test this, we formed HVSC and CMPC spheroids and encapsulated them in B0.25M2.25 hydrogels with or without UPy‐cRGD additives. Strikingly, within 1 day after encapsulation, HVSCs started to migrate from the spheroids toward the hydrogel matrix when UPy‐cRGD molecules were incorporated in the gel compositions (**Figure** **6**a). After 7 days, cell migration toward the matrix was significant for both spheroid types and was further enhanced at Day 14 for UPy‐cRGD containing groups (Figure 6a–c). In contrast, no HVSC migration was detected and CMPC spheroids slightly shrank during the culture period when hydrogels did not contain UPy‐cRGD. Importantly, the majority of the cells remained viable for both spheroid types after 14 days of culture in hydrogels with or without UPy‐cRGD additives (Figure 6c).

Removal of spheroids and organoids from their culture matrix is of remarkable importance for their thorough characterization and therapeutic applications. Therefore, after 14 days, we extracted the spheroids from the hydrogels by disrupting the gel network via gentle mechanical shearing using pipette tips. The extracted spheroids were seeded onto glass slides and were imaged after 2 days of culture. Confocal images showed that cells adhered and spread out on the glass slides, and migrated from the spheroids onto the substrate surfaces (Figure 6d), revealing that they remained functional within the spheroids during the 14 days culture period.

To further evaluate the applicability of this dynamic material system for 3D culture, we next encapsulated human liver hepatocyte organoids in the supramolecular hydrogels and monitored their growth over 7 days of culture in proof‐of‐concept experiments (Figure S15, Supporting Information). The organoids encapsulated in B0.25M2.25 hydrogels displayed high levels of ATP production and their surface area doubled during the culture period. Moreover, a budding‐like morphology^[^ ^53^ ^]^ emerged at the organoid periphery when UPy‐cRGD additives were included in the hydrogel composition, indicating the ability of our supramolecular hydrogels to serve as a modular platform for facilitating organoid culture.

## 3 Conclusions

In conclusion, supramolecular hydrogels based on combinations of slow and fast exchanging molecules have shown to function as synthetic ECM mimics for diverse cell culture schemes, ranging from single cells to spheroids, and organoids. Effective incorporation and presentation of biochemical cues in these materials is a predominant requirement for their biomedical translation and their use as a synthetic alterative to the ill‐defined and less tunable Matrigel.^[^ ^54^ ^]^

This work demonstrates the importance of molecular exchange dynamics on effective functionalization of supramolecular hydrogels with adhesive ligands. Previous investigations have shown enhanced spreading of cells cultured on or within more quickly relaxing hydrogels that were covalently tethered with RGD ligands.^[^ ^26^, ^55^ ^]^ Consequently, an intuitive expectation would suggest that for hydrogels containing adhesive ligands, a more dynamic behavior would result in enhanced cell adhesion and spreading. Nevertheless, our results reveal that adhesive ligands are ineffective in supramolecular hydrogels with excessively dynamic behavior, but dampening the exchange dynamics can render these materials cell‐adhesive. The lack of cell adhesion and spreading for the more dynamic hydrogels in this study likely arises from a low binding energy, and hence high binding/unbinding rate, of the UPy‐cRGD additives within the supramolecular fibers, thereby not allowing for the engagement of “molecular clutches” that drive mechanotransduction.^[^ ^56^ ^]^ The tuning of molecular exchange dynamics can be achieved by altering the molecular design and the ratio between the supramolecular building blocks employed for hydrogel formation.

3D encapsulation of cells in supramolecular hydrogels was previously realized in two‐component systems containing complementary domains.^[^ ^57^, ^58^ ^]^ The current study presents a strategy to employ non‐gel‐forming regimes of supramolecular building blocks with self‐complementary interactions for 3D encapsulation of cells, spheroids, and organoids at physiological conditions. Previous work has indicated that cell spreading in dynamic hydrogels is not largely determined by the tethering of adhesive ligands, whereas early protein deposition and remodeling were found to play an essential role in this process.^[^ ^27^ ^]^ However, our results demonstrate that upon optimization of the hydrogels’ dynamic profile, adhesive ligand tethering can drive cellular adhesion and spreading, as well as cell–matrix interactions in multicellular spheroids and organoids. These results, however, do not undermine the critical role that nascent proteins can play at later time points, and the potential interplay between tethered biophysical cues and nascent protein deposition in long term cultures remains to be studied. Moreover, when comparing different systems, it is important to consider that several other factors involved in their design can also impact the effects of their tethered bioactive cues as well as other aspects of their biological performance. These factors include the type of polymer used (natural vs synthetic) as well as the number of possible cross‐links per polymer chain.

Finally, the modularity of the supramolecular assembly strategy utilized here offers a substantial advantage for on‐demand variation of other hydrogel properties such as stress relaxation, which are important for directing cellular behavior. The strategy described here also paves the way for effective tethering of additional biochemical cues into supramolecular hydrogels, enabling the modulation of signaling pathways and the regulation of cell proliferation and differentiation.

## 4 Experimental Section

### Synthesis and Characterization of Supramolecular Molecules

UPy‐PEG‐UPy molecules were synthesized as described previously.^[^ ^30^ ^]^ UPy‐G, UPy‐cRGD, and UPy‐Cy5 were synthesized and characterized as described in the Supporting Information.

Partition coefficient (log*P*) values were calculated with MarvinSketch 20.11 software using the consensus mode.

### Cryogenic Transmission Electron Microscopy

Stock dispersions (10 mg mL^−1^) of supramolecular fiber assemblies were prepared by dissolving B‐ and M‐type molecules at different ratios in alkaline PBS solutions (containing 80 mm of NaOH), followed by the addition of HCl (final concentration = 83 mm) for pH neutralization. Thereafter, Cryo‐TEM sample preparation was done using dispersions with concentrations ranging from 0.05 to 10 mg mL^−1^ for optimal fiber visibility. Lacey carbon film grids (200 mesh, 50 µm hole size; Electron Microscopy Sciences) were surface plasma treated at 5 mA for 40 s using a Cressington 208 carbon coater, and each dispersion (3 µL) was applied onto each grid. Using an automated vitrification robot (FEI Vitrobot Mark III), excess sample was removed through blotting with filter paper for 3 s at −3 mm. Thin films of dispersions were vitrified by plunging the grids into liquid ethane just above its freezing point. Imaging was carried out on a FEI‐Titan TEM equipped with a field emission gun operating at 300 kV. Samples were imaged using a post‐column Gatan energy filter and a 2048 × 2048 Gatan CCD camera. Micrographs were recorded at low dose conditions, using a defocus setting of −10 µm at 25000× magnification, or defocus setting of −40 µm at 6500× magnification. Contrast and brightness of images were manually adjusted using the ImageJ software to improve the visibility of fibers.

### Preparation of Hydrogels

*pH‐induced gelation*: B‐ and M‐type molecules were dissolved at 70 °C in an alkaline PBS solution. The PBS solution contained 80 or 160 mm NaOH for the preparation of hydrogels with ≤5 or 10 wt% polymer contents, respectively. Thereafter, to initiate gelation, a specific volume of 1 m HCl solution was added to the alkaline solution of building blocks and additives to reach neutral pH. The resulting mixture contained 83 or 113 mm of HCl for the hydrogels with ≤5 or 10 wt% polymer contents, respectively. The hydrogels were kept overnight in a 4 °C fridge to ensure complete gelation.

*Mixing‐induced gelation*: B‐ or M‐type molecules were dissolved separately at 70 °C in alkaline PBS solutions (containing 80 mm NaOH). Thereafter, a specific volume of HCl solution (1 m) was added to each solution at room temperature to reach neutral pH (final HCl concentration = 83 mm). To initiate gelation, the resulting B and M dispersions were mixed via pipetting.

### Rheological Characterizations

A Discovery hybrid rheometer (DHR‐3, TA Instruments) was used for rheological characterizations of supramolecular solutions and hydrogels. Hydrogel disks were made via the pH‐induced gelation method inside cylindrical Teflon molds (diameter = 8 mm, height = 2 mm). Pre‐formed hydrogel disks were analyzed using a flat stainless‐steel geometry (diameter = 8 mm) at a gap height of 0.5–2 mm. Low viscosity silicon oil (47 V 100, RHODORSIL) was applied to seal the gap around the hydrogel disks to minimize drying during the measurements at 37 °C. Non‐gelling samples were tested at 37 °C (unless otherwise indicated) using flat stainless‐steel (diameter = 8 mm) or 2.007° cone‐plate aluminum (diameter = 20 mm, with solvent trap to minimize sample drying) geometries at gap heights of 500 or 56 µm, respectively. Mixing‐induced gelation was evaluated using the cone‐plate geometry at a gap height of 56 µm by mixing the dispersions on the Peltier plate using a pipette immediately prior to the measurements. Strain sweep measurements (1–1000% strain, 1 rad s^−1^) were performed to determine the linear viscoelastic region of hydrogels. Frequency sweeps were carried out with frequencies ranging from 100 to 0.01 rad s^−1^, at a constant strain of 1%. Time sweeps were carried out at a constant frequency and a constant strain of 1 rad s^−1^ and 1%, respectively. Stress relaxation experiments were performed by applying a strain of 1%, and monitoring the generated stress for 10 min. The data were normalized using the stress detected at 1 s for each sample.

### Fluorescence Recovery after Photo‐Bleaching

FRAP measurements were carried out using a Leica TCS SP5 inverted confocal microscope (Leica Microsystems) equipped with a 20× objective (HCX PL APO CS 20.0 × 0.70 DRY UV). Hydrogels were formed through pH‐induced gelation inside the cylindrical chamber (diameter = 7 mm) of 35‐mm dishes with cover glass bottoms (MatTek, Ashland, MA). Hydrogels contained 20 µm of UPy‐Cy5 or 0.5 mg mL^−1^ of FITC‐Dextran (Fluorescein isothiocyanate–dextran; average MW 20, 100, or 2000 kDa; Sigma‐Aldrich) for exchange dynamics and pore size measurements, respectively. To minimize sample drying during the measurements, the chamber was covered with a cover glass and sealed with nail polish, wet tissue paper was placed in the dish, and the lid was sealed with Parafilm. Prior to each measurement, the sample was placed inside the environmental chamber of the microscope at 37 °C to equilibrate for 1 h. Exchange dynamics experiments were carried out via sample illumination using white laser at 646 nm wavelength for Cy5 excitation. Emission was collected at 660–700 nm wavelength using a hybrid detector. A circular area with a diameter of 20 µm was photo‐bleached at 60% laser power for 31 frames (0.653 frame s^−1^), and the post‐bleaching time‐lapse imaging was performed for >12 h. Data normalization was conducted by dividing the fluorescence intensity in the bleached area by the fluorescence intensity in a non‐bleached circular area of same size in each image. τ_1/2_ and mobile fraction were determined using the easyFRAP software^[^ ^59^ ^]^ by means of a double exponential fitting. The initial rate of recovery was determined by calculating the slope of the linear regression fit of the recovery curve for the first 60 s post‐bleaching.

Experiments concerning pore size evaluation were carried out via sample illumination using white laser at 493 nm wavelength for FITC excitation. Emission was collected at 520 nm wavelength using a hybrid detector. A circular area with a diameter of 20 µm was photo‐bleached at 60% laser power for 15 frames (0.653 frame s^−1^), and the post‐bleaching time‐lapse imaging was performed for 5 min. Data normalization was performed as described above, and the initial rate of recovery was calculated for the first 2 s post‐bleaching.

### Cell Culture

HVSCs were harvested from the human vena saphena magna based on previously established protocols.^[^ ^60^, ^61^ ^]^ To this end, guidelines regarding the secondary use of patient material were employed based on the Dutch code of conduct for responsible use of patient material. Review by a Medical Ethics Examination Committee was not required according to the Dutch medical scientific research with human subjects act (WMO) for secondary use of patient material. These HVSCs had previously been characterized as myofibroblasts by immunohistochemistry using the primary antibodies of anti‐vimentin, anti‐desmin, and anti‐α‐smooth muscle actin.^[^ ^62^ ^]^ HVSC expansion and culture was performed in Dulbecco's modified Eagle's medium (Gibco) supplemented with 10% fetal bovine serum (FBS; Greiner Bio‐One), 1% GlutaMax (Gibco), and 1% penicillin/streptomycin (Lonza).

CMPCs were isolated by enzymatic digestion of harvested human fetal cardiac tissues and magnetic bead sorting of cells using anti‐Sca‐1 microbeads (Miltenyi Biotech) as described previously,^[^ ^63^ ^]^ following approval by the Medical Ethics committee of the University Medical Center Utrecht and in accordance with the Declaration of Helsinki. Subsequently, L9TB CMPCs were immortalized through lentiviral transduction of hTERT and BMI‐1, as reported previously.^[^ ^64^ ^]^ CMPC expansion and culture was performed in SP++ growth medium, composed of a 3:1 volumetric mixture of M199 (Gibco) and EGM‐2 BulletKit (Lonza), supplemented with 10% FBS (Greiner Bio‐One), 1% non‐essential amino acids (Gibco), and 1% penicillin/streptomycin (Lonza). CMPC expansion was carried out in gelatin‐coated flasks. For all experiments, the culture medium was refreshed every 3 days. Prior to cell culture, pre‐formed supramolecular hydrogels (for 2D culture) or dispersions (for 3D culture) were UV‐treated for 15 min as a precautionary disinfection step.

2D cell culture experiments were carried out by forming the hydrogels (70 µL) through pH‐induced gelation inside 96‐well cell culture plates. Prior to cell seeding, the hydrogels were incubated with culture medium (≈200 µL) for 15 min to ensure physiological pH and ionic concentration during the cell culture. Thereafter, the medium was removed, cell suspension (200 µL) containing 250000 cells (1.25 million cells mL^–1^) were added into each well, and the plates were incubated (37 °C, 5% CO_2_) for 1 or 3 days. At each time point, the medium was removed and the samples were washed with PBS to remove non‐adherent cells.

3D cell culture experiments were carried out by encapsulation of cells in hydrogels (500000 cells mL^−1^) using the mixing‐induced gelation method. To this end, cells were included in the supramolecular dispersion containing B‐type molecules (B dispersion). Thereafter, the M dispersion (50 µL) was mixed with the B dispersion (50 µL; +cells) inside each well of 8‐well chambered cover glasses using a pipette. The resulting mixtures were kept in an incubator for 15 min for completion of the gelation step. Thereafter, culture medium (≈200 µL) was added into each well and the cells were cultured for up to 7 days. To block exocytosis, 120 nm of EXO‐1 (Sigma Aldrich) was added to culture media and replenished daily. To inhibit local matrix metalloproteinase activity, 5 nm of recombinant TIMP‐3 (R&D Systems) was encapsulated in the hydrogels, added to culture media, and replenished daily.

### Quantification of Number of Adhered Cells

The number of cells adhered to the surface of hydrogels was quantified using a CyQuant Cell Proliferation Assay (Invitrogen), following the manufacturer's guideline. The assay measures the DNA content in cell lysates by utilizing a dye that displays strong fluorescence enhancement upon binding to nucleic acids. A standard curve was plotted using known cell concentrations, which was used to translate the fluorescence intensity to cell number for each sample.

### Cell Staining and Imaging

Actin cytoskeleton and nuclei staining were performed using phalloidin‐FITC and 4′,6‐diamidino‐2‐phenylindole (DAPI), respectively. Prior to staining, the cells were fixated with 3.7% formaldehyde, washed twice with PBS, and permeabilized with 0.5% Triton X‐100. Live/Dead staining was carried out according to the manufacturer's protocol (Thermo Fisher Scientific) using calcein‐AM and propidium iodide to stain for live and dead cells, respectively. A Leica TCS SP5 inverted confocal microscope (Leica Microsystems) was used to acquire z‐stack images using 10× (HCX PL APO CS 10.0 × 0.40 DRY UV) and 63× (HCX PL APO CS 63.0 × 1.20 WATER UV) objectives.

Immunohistochemical staining for YAP quantification experiments were carried out by first washing the cells twice with PBS, followed by fixation with 3.7% formaldehyde. Thereafter, the cells were washed thrice with PBS and permeabilized using a blocking solution (10% donkey serum + 0.3% Triton X‐100 in PBS). Next, the cells were incubated with anti‐YAP1 antibody (1:100 dilution, Santa Cruz sc‐101199) in the blocking solution overnight at 4 °C. Thereafter, the cells were washed thrice with PBS containing 0.3% Triton X‐100 and incubated with phalloidin (1:200 dilution) and anti‐mouse IgG Alexa488‐conjugated antibody (1:400, Jackson ImmunoResearch 715‐545‐150) for 1 h. Finally, the cells were stained with DAPI at a dilution of 1:500 for 10 min, washed thrice with PBS, and imaged using a Leica TCS SP8 X inverted confocal microscope (Leica Microsystems).

### Cell Morphology, Yes‐Associated Protein Translocation, and Viability Analyses

Cell morphology was analyzed from maximum‐intensity z‐projections of images obtained after actin cytoskeleton and nuclei staining. To this end, ImageJ software was used to determine the circularity and the length of the longest axis of individual cells. The cell circularity was calculated as 4π multiplied by the cell area, then divided by the square of cell perimeter.

To determine the localization of YAP, maximum‐intensity z‐projections of images were obtained from channels corresponding to DAPI, phalloidin, and anti‐YAP antibody staining. Subsequently, ImageJ software was used for overlaying the channels and thresholding. The overlayed images were used to determine the cytoplasmic and nuclear areas, after which the average YAP intensity was determined in those respective areas using the “measure” function in ImageJ. The ratio between the concentration of YAP present in nuclear and cytoplasmic regions of cells was then used as a measure of YAP nuclear translocation.

Cell viability in 2D culture experiments was determined by measuring the lactate dehydrogenase released from cells with a damaged plasma membrane using CytoTox‐ONE assay (Promega), following the assay manufacturer's guidelines. Cell viability in 3D culture experiments was calculated by counting live (green) and dead (red) cells in maximum‐intensity z‐projections of microscopy images.

### Spheroid Formation and Culture

*Spheroid formation*: Cell suspensions were prepared at a concentration of 25000 cells mL^−1^. Cell suspension (200 µL) was added into each well of non‐adhesive round bottom 96‐well plates (Nunclon Sphera, Thermo Fisher Scientific). The plates were centrifuged for 2 min at 200 RCF, and incubated (37 °C, 5% CO_2_) for 5 days for spheroid formation. Thereafter, the spheroids were collected into Eppendorf tubes using a pipette. The spheroid density in medium was adjusted to 360 spheroids mL^−1^ through centrifugation for 2 min at 300 RCF.

*Spheroid encapsulation*: The spheroid encapsulation was carried out through the mixing‐induced gelation method. To this end, the spheroids were included in B dispersions. Thereafter, the M dispersion was mixed with a B dispersion (+spheroids) inside an Eppendorf tube through gentle pipetting for ≈30 s. The mixtures (100 µL) were then pipetted onto 8‐well chambered cover glasses, and placed in an incubator (37 °C, 5% CO_2_) for 20 min for completion of the gelation step. Thereafter, culture medium (≈200 µL) was added into each well, and the spheroids were cultured for up to 14 days. The culture medium was refreshed every 3 days.

*Spheroid imaging, staining, and extraction*: A phase contrast microscope (Invitrogen EVOS XL Digital Inverted Microscope) was used to image the spheroids at different time points. At Day 14, Live/Dead staining was carried out according to the manufacturer's protocol (Thermo Fisher Scientific) using calcein‐AM and propidium iodide, and the spheroids were imaged using a Leica TCS SP5 inverted confocal microscope (Leica Microsystems). Thereafter, the spheroids were extracted from the hydrogels through gentle mechanical disruption of the gel networks using pipette tips. The extracted spheroids were seeded onto 8‐well chambered cover glasses, and cultured (37 °C, 5% CO_2_) for 2 days with culture medium (≈200 µL). Thereafter, Live/Dead imaging was carried out using the inverted confocal microscope.

*Quantification of cell migration distance*: The cell migration was quantified by measuring the average distance that the cells migrated from the initial surface of spheroids (at Day 0 of encapsulation) into the surrounding hydrogel matrix. To this end, phase contrast microscopy images of spheroids were analyzed using ImageJ software. Accordingly, the longest distance between any two points (Feret's diameter; *D*_F_) of the objects composed of the spheroids and their migrating cells was quantified. The cell migration distance from individual spheroids at each time point was calculated as *D*_F_ minus *D*_F(Day 0)_, divided by two.

### Statistics

Statistical analyses were carried out using GraphPad Prism 8 software. Statistical comparisons between experimental groups were made using one‐way or two‐way analysis of variance followed by Bonferroni post hoc test. Figure captions describe the statistical tests employed for each experiment and the notations used to indicate statistical differences.

## Conflict of Interest

The authors would like to disclose a submitted patent application related to the supramolecular hydrogels reported in this manuscript. International Patent Application filed with the European Patent Office on July 13, 2020 by the Eindhoven University of Technology (Priority date: July 12, 2019), with PCT Application No. PCT/EP2020/069787. Inventors: M.D., S.S., and P.Y.W.D.

## Author Contributions

M.D. designed the study, performed the experiments, and wrote the manuscript with contributions from other authors. S.S. contributed to the study design and co‐performed the cell and spheroid culture experiments. J.F.v.S. contributed to the design, data acquisition, and analysis of YAP translocation experiments. D.J.W. contributed to the design, data acquisition, and analysis of FRET experiments. S.I.S.H. contributed to the study design and performed the organoid culture experiments. M.M.C.B. contributed to the study design and the manuscript content. M.J.G.S. synthesized UPy‐Cy5 additives, and co‐performed FRAP and Cryo‐TEM experiments. F.J.M.H. synthesized supramolecular building blocks and additives. H.M.J. contributed to the design of supramolecular building blocks and additives. P.Y.W.D. conceived and supervised the study, and edited the manuscript.

## Supporting information

Supporting Information

## Data Availability

Research data are not shared.
