# Supplementary material for: Engineering the Dynamics of Cell Adhesion Cues in Supramolecular Hydrogels for Facile Control over Cell Encapsulation and Behavior
Source: Adv Mater. 2021 Aug 1;33(37):2008111. doi: 10.1002/adma.202008111 (PMC11468203; doi:10.1002/adma.202008111)
Supplement: Supplementary file 1 — Supporting Information [file ADMA-33-2008111-s001.pdf]

© 2021 Wiley-VCH GmbH

ADVANCED MATERIALS

Supporting Information

for *Adv. Mater.,* DOI: 10.1002/adma.202008111

Engineering the Dynamics of Cell Adhesion Cues in Supramolecular Hydrogels for Facile Control over Cell Encapsulation and Behavior

*Mani Diba, Sergio Spaans, Simone I. S. Hendrikse, Maartje M. C. Bastings, Maaike J. G. Schotman, Johnick F. van Sprang, Dan Jing Wu, Freek J. M. Hoeben, Henk M. Janssen, and Patricia Y. W. Dankers**

Supporting Information

Engineering the Dynamics of Cell Adhesion Cues in Supramolecular Hydrogels for Facile Control Over Cell Encapsulation and Behavior

*Mani Diba, Sergio Spaans, Simone I. S. Hendrikse, Maartje M. C. Bastings, Maaike J. G. Schotman, Johnick F. van Sprang, Dan Jing Wu, Freek J. M. Hoeben, Henk M. Janssen, and Patricia Y. W. Dankers**

Supplementary Table:

Table S1. Overview of sample IDs and molar ratios of components present in different 5 wt% formulations.

B-type molecules (UPy-PEG-UPy)	M-type molecules (UPy-G ± UPy-cRGD/UPy-Cy5)	Molar Ratio ID	Concentration of UPy moieties (mM)	Sample ID
5 wt%	0 wt%	B	9	B5
4.5 wt%	0.5 wt%	B_1M_1	12	B4.5M0.5
3.5 wt%	1.5 wt%	B_1M_4	19	B3.5M1.5
2.5 wt%	2.5 wt%	B_1M_9	25	B2.5M2.5
1.5 wt%	3.5 wt%	$B_1M_{22(21*)}$	32	B1.5M3.5
0.5 wt%	4.5 wt%	$B_1M_{84(81*)}$	39	B0.5M4.5
0 wt%	5 wt%	M	42	M5

*For hydrogels containing 3 mM of UPy-cRGD, due to the difference between molecular weights of UPy-G and UPy-cRGD.

Supplementary Figures:

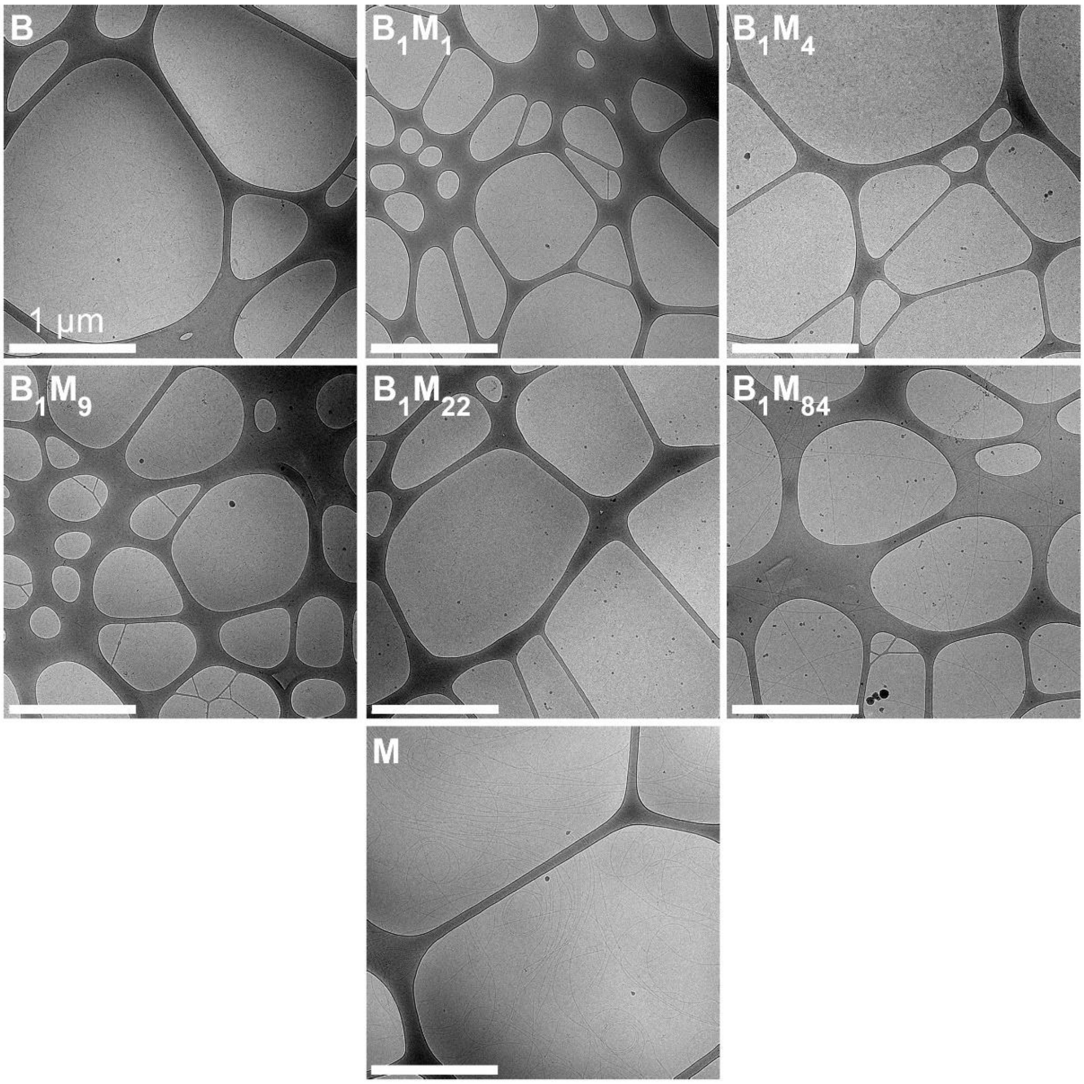

Figure S1. Overview of cryo-TEM images showing the morphology of fibers assembled from M- and B-type molecules at different molar ratios.

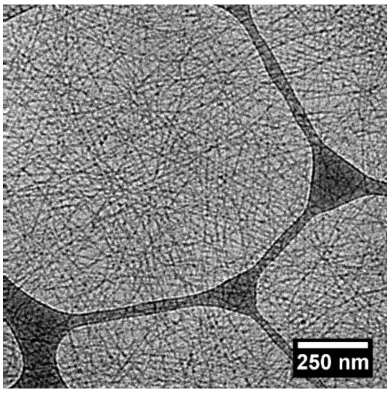

Figure S2. Cryo-TEM image showing the percolation of B_1M_{84} fibers at a building block concentration of >1 mg mL^{-1}.

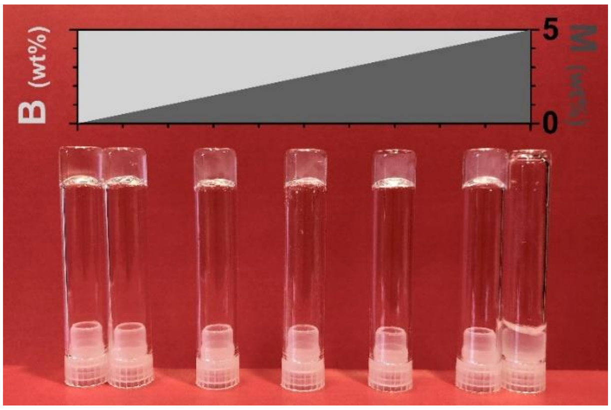

Figure S3. Digital photograph showing different supramolecular compositions subjected to the inverted-vial test after 24 h of gelation. The inset graph indicates the composition of each sample.

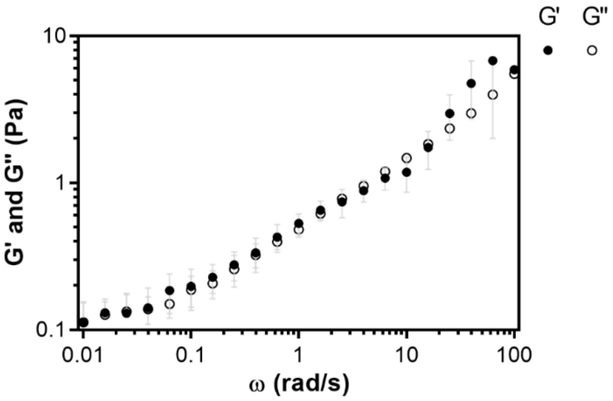

Figure S4. Frequency dependence of storage (G′) and loss (G″) moduli of supramolecular samples with M5 composition. Data are shown for n=3 independent tests, and as mean ± s.d.

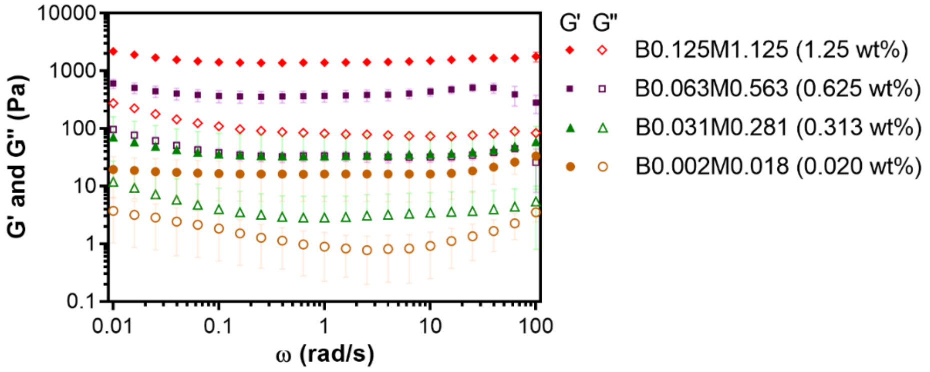

Figure S5. Frequency dependence of viscoelastic behavior of hydrogels with different total polymer concentrations with a fixed molar ratio of B_1M_{84}, measured at 1% strain. All values are presented as mean ± s.d. for n=3 per experimental group.

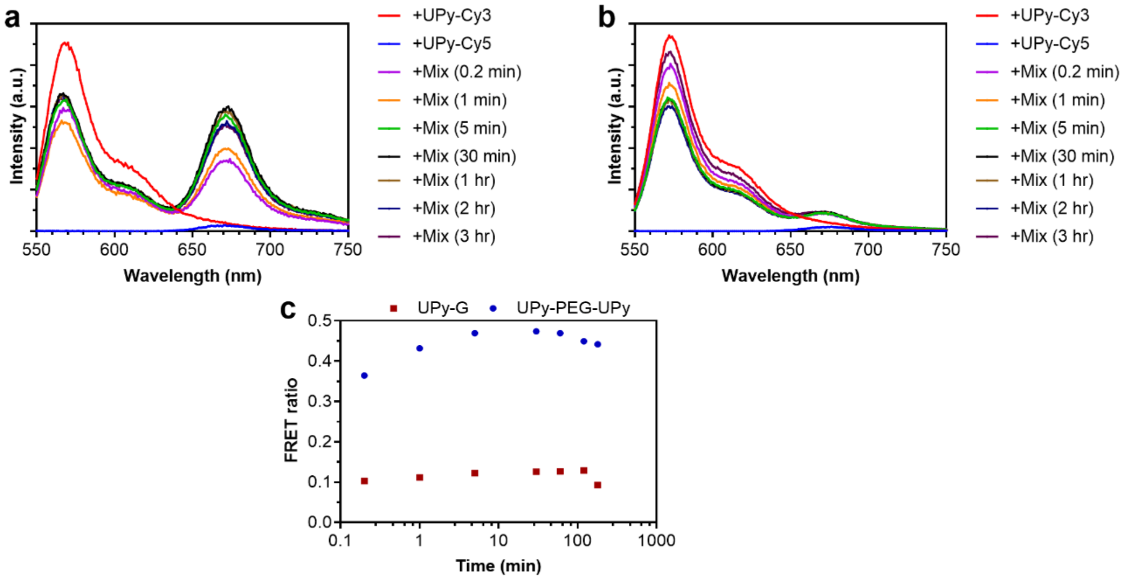

Figure S6. Emission scans obtained at different time points after mixing (Mix) two dispersions of **a**, UPy-PEG-UPy or, **b**, UPy-G supramolecular fibers with different dyes. Control groups labeled as UPy-Cy3 and UPy-Cy5 in the graphs contained only one type of UPy-dye additive. **c**, FRET ratios calculated based the data presented in **a** and **b** after the mixing step.

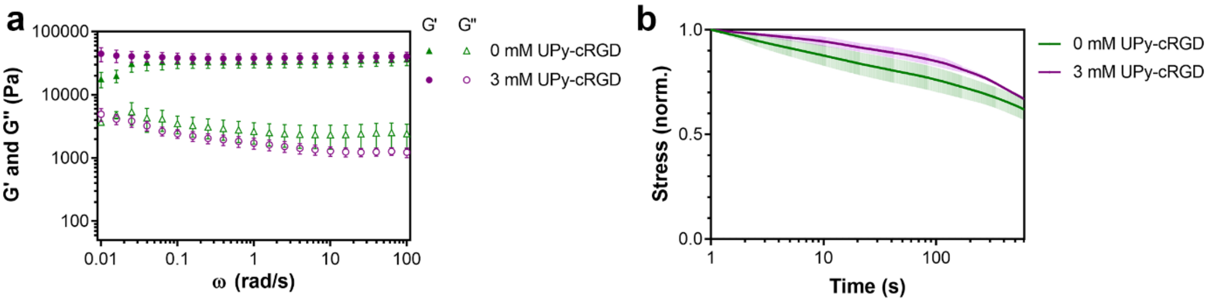

Figure S7. a, Frequency dependence of viscoelastic behavior of B0.5M4.5 hydrogels with or without UPy-cRGD additives, measured at 1% strain. **b**, Stress relaxation behavior of B0.5M4.5 hydrogels, with or without UPy-cRGD additives, measured by subjecting the hydrogels to 1% strain. All values are presented as mean ± s.d. for n=3 per experimental group.

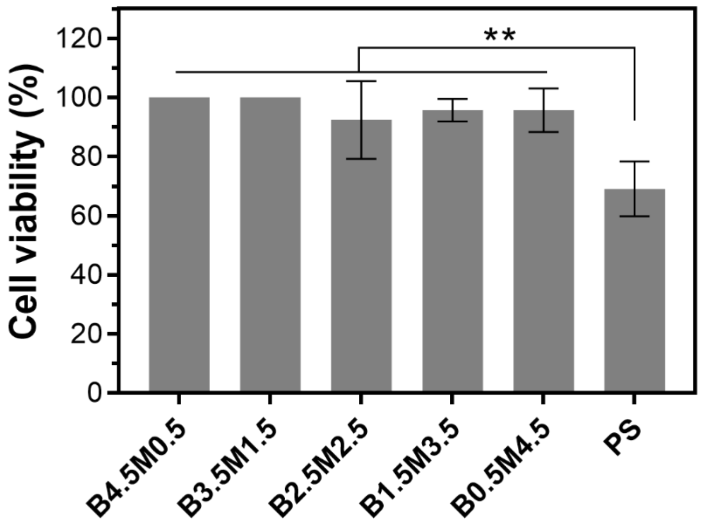

Figure S8. Percentage viability of HVSCs after 3 days of culture on different supramolecular hydrogel compositions. PS indicates polystyrene control. All hydrogels contained 3 mM of UPy-cRGD additives. **, $p<0.01$; one-way analysis of variance (ANOVA) followed by Bonferroni post hoc. Results were obtained from 4 biologically independent experiments per group, and all values are shown as mean ± s.d.

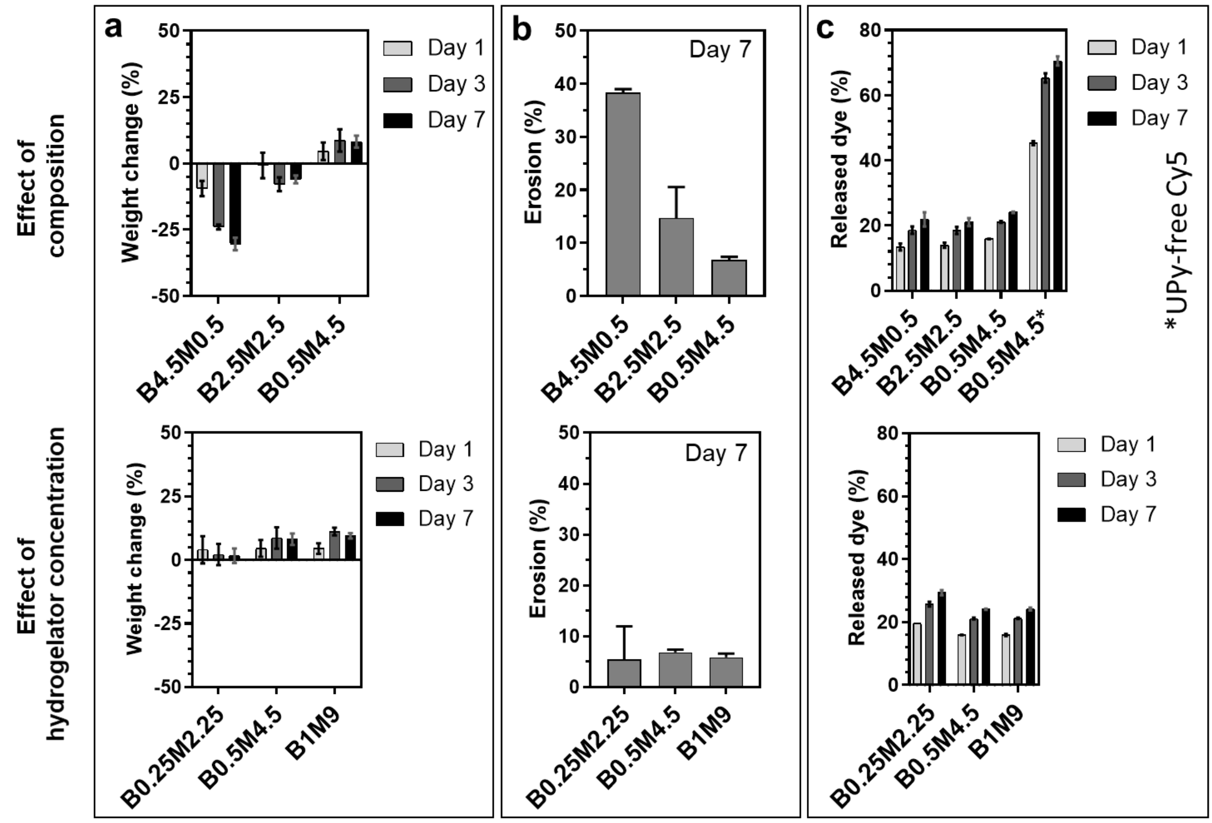

Figure S9. **a**, Weight change, **b**, erosion and, **c**, UPy-Cy5 or Cy5 additive release from the supramolecular hydrogels during an immersion test at 37 °C. Hydrogels contained 100 μM of UPy-Cy5 or Cy5 additives. All values are presented as mean ± s.d. for n=3 per experimental group.

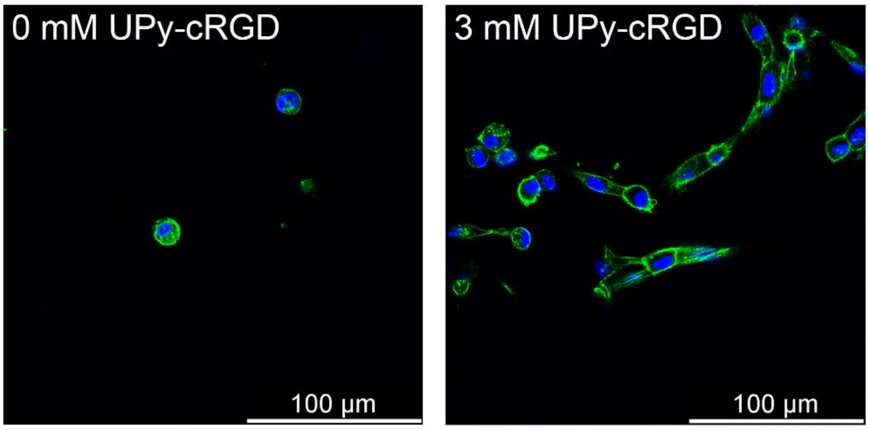

Figure S10. Representative images showing CMPCs after 1 day of culture on M0.5B4.5 hydrogels without or with UPy-cRGD additives. Green and blue colors in images indicate actin and nucleus staining, respectively.

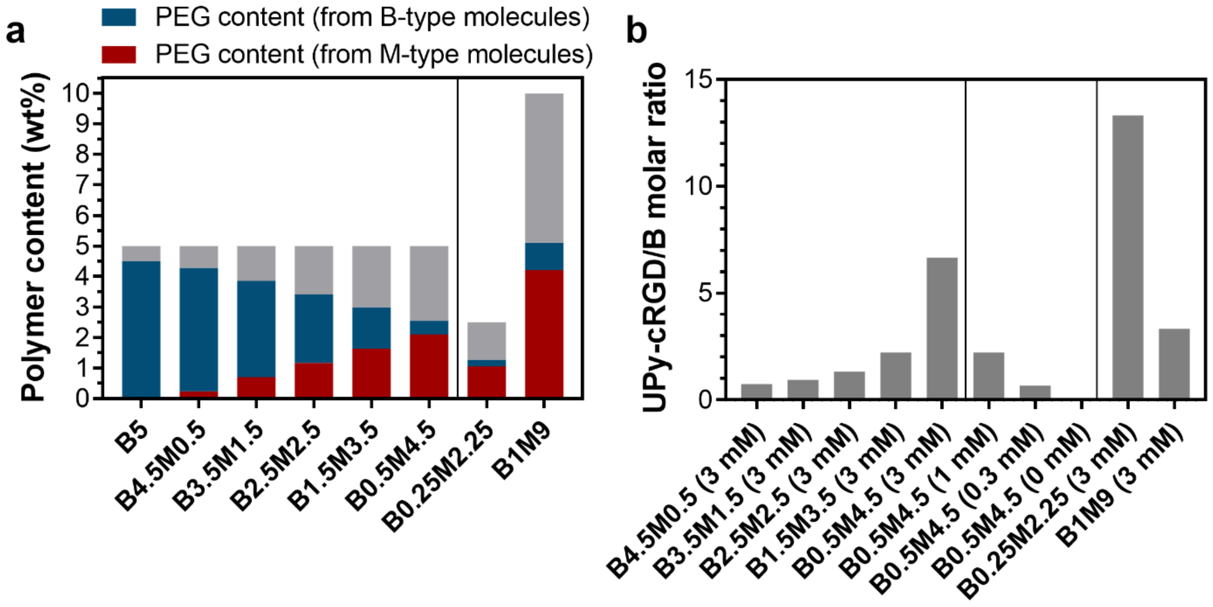

Figure S11. a, Estimated PEG content of hydrogels with different compositions. **b,** Molar ratios between UPy-cRGD additives and B-type molecules (UPy-cRGD/B) in different hydrogel compositions. Values within parentheses indicate the molar content of the UPy-cRGD additive in each composition.

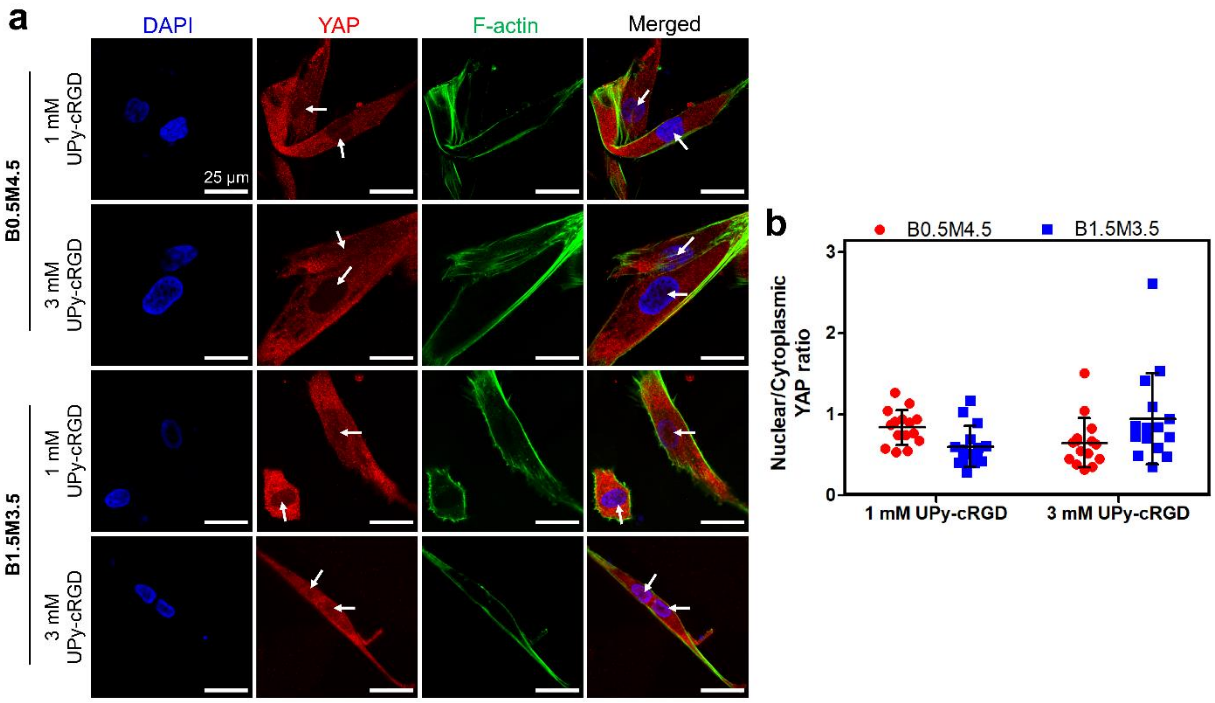

Figure S12. a, Representative images of HVSCs upon immunofluorescence staining for nucleus (blue), YAP (red), and actin (green) after 1 day of culture on supramolecular hydrogels with different compositions and UPy-cRGD concentrations. Arrows indicate the nuclei. **b**, Quantification of the nuclear/cytoplasmic ratio of the YAP concentration in cells after 1 day of culture.

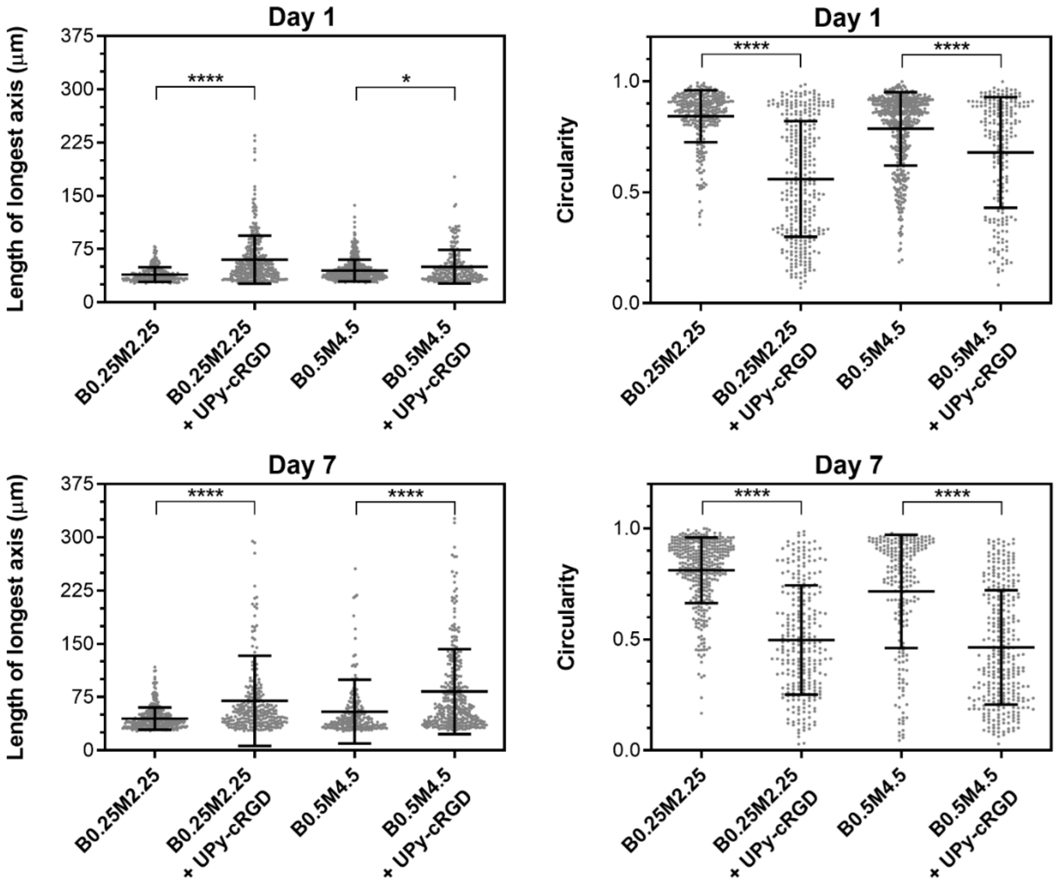

Figure S13. Length of longest axis and circularity of HVSCs encapsulated in supramolecular hydrogels without or with 3 mM of UPy-cRGD additives, after 1 or 7 days of culture. *, $p<0.05$; ****, $p\leq0.0001$; one-way analysis of variance (ANOVA) followed by Bonferroni post hoc. Results were obtained from 3 biologically independent experiments per group, and all values are shown as mean ± s.d. All results were obtained from 3 independent experiments per group, and their values are shown as mean ± s.d. Data points represent features of individual cells, with n comprising the total number of cells per group that were detectable/analyzed among 3 experiments.

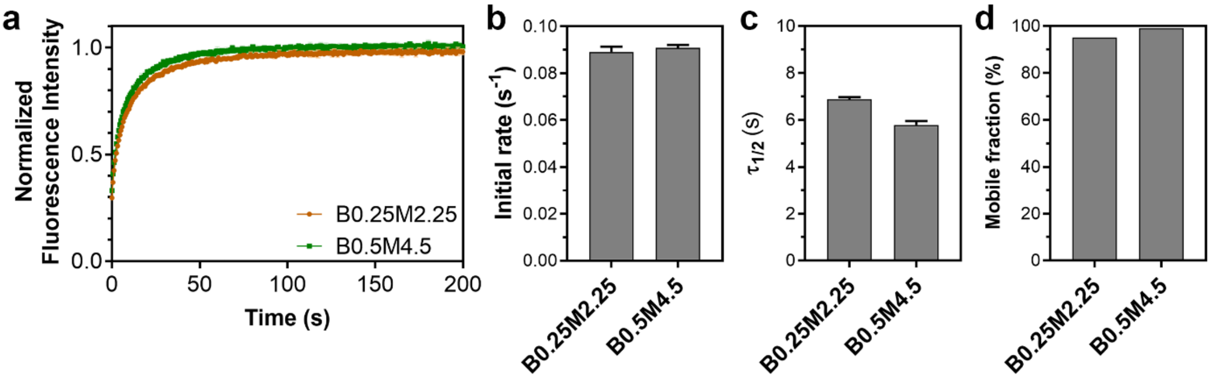

Figure S14. **a**, Fluorescence recovery after photo-bleaching (FRAP) tests performed on supramolecular hydrogels containing 0.5 mg mL^{-1} of FITC-Dextran (MW 2000 kDa). **b**-**d**, Quantified FRAP results showing (**b**) the rate of fluorescence recovery during the first 2 s after photo-bleaching (Initial rate), (**c**) the timespan during which the fluorescence intensity recovers to half its mobile fraction ($\tau_{1/2}$), and (**d**) the fraction of fluorescence intensity that recovers when fluorescence intensity curves reach plateau values (Mobile fraction). All values are presented as mean ± s.d. for n=3 per experimental group.

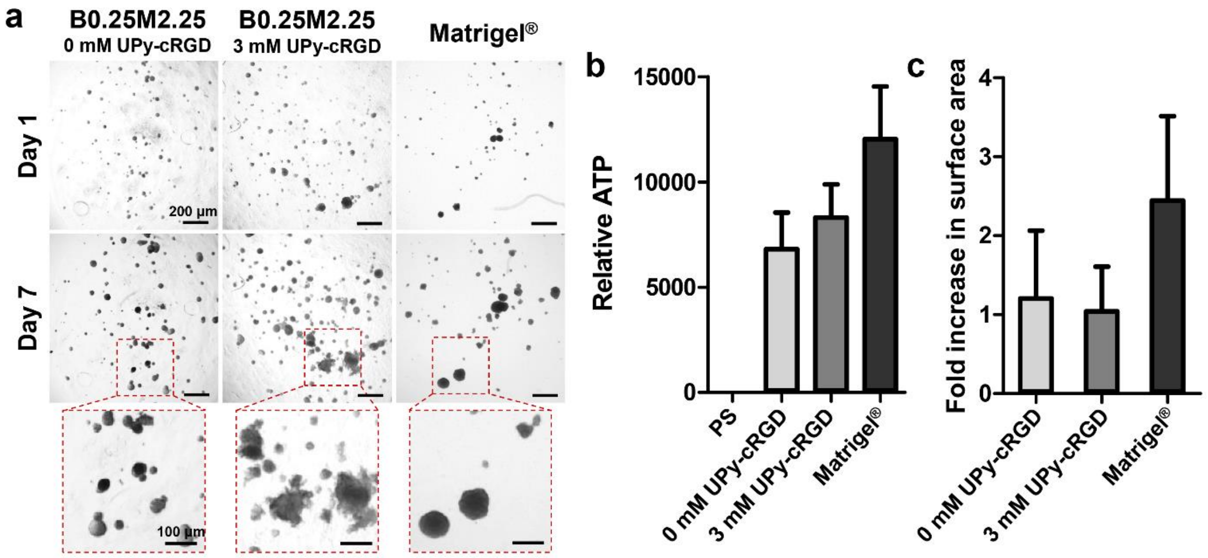

Figure S15. Organoid encapsulation and spreading in supramolecular hydrogels. **a**, Representative optical microscopy images showing the morphology of hepatic liver organoids encapsulated in different hydrogels, upon culture for 1 and 7 days. **b**, Relative ATP level of the organoids upon 7 days of culture within different hydrogels. PS indicates the polystyrene control. **c**, Fold increase in surface area of the organoids from day 1 to day 7 of the culture period.

Supplementary Methods:

Supplementary Method 1: Synthesis of UPy-G and UPy-cRGD

Materials:

All reagents, chemicals, materials and solvents were obtained from commercial sources and were used as received. All solvents were of AR quality. In the synthesis procedures, equivalents (eq) are molar equivalents. ^{1}H-NMR spectra were recorded on a Bruker Avance™ III HD spectrometer at 298 K (400 MHz for ^{1}H-NMR). Chemical shifts are reported in ppm downfield from TMS at room temperature. Abbreviations used for splitting patterns are s = singlet, t = triplet, q = quartet, m = multiplet and br = broad. HPLC-PDA/MS was performed using a Shimadzu LC-10AD VP series HPLC coupled to a diode array detector (Finnigan Surveyor PDA Plus Detector, Thermo Electron Corporation) and an ion trap mass spectrometer (LCQ Fleet, Thermo Fisher Scientific). HPLC analyses were performed using a Alltech® Alltima™ HP C_{18} 3μm column using an injection volume of 1-4 μL, a flow rate of 0.2 mL min^{-1} and a gradient (5% to 100% in 10 min, then held at 100% for 3 min) of MeCN in H_2O (both containing 0.1 % formic acid) at 298 K. Preparative RP-HPLC (MeCN / H_2O with 0.1 v/v% formic acid) was performed using a Shimadzu SCL-10A VP coupled to two Shimadzu LC-8A pumps and a Shimadzu SPD-10AV VP UV-Vis detector on a Phenomenex Gemini® 5 μm C_{18} 110A column.

Synthesis:

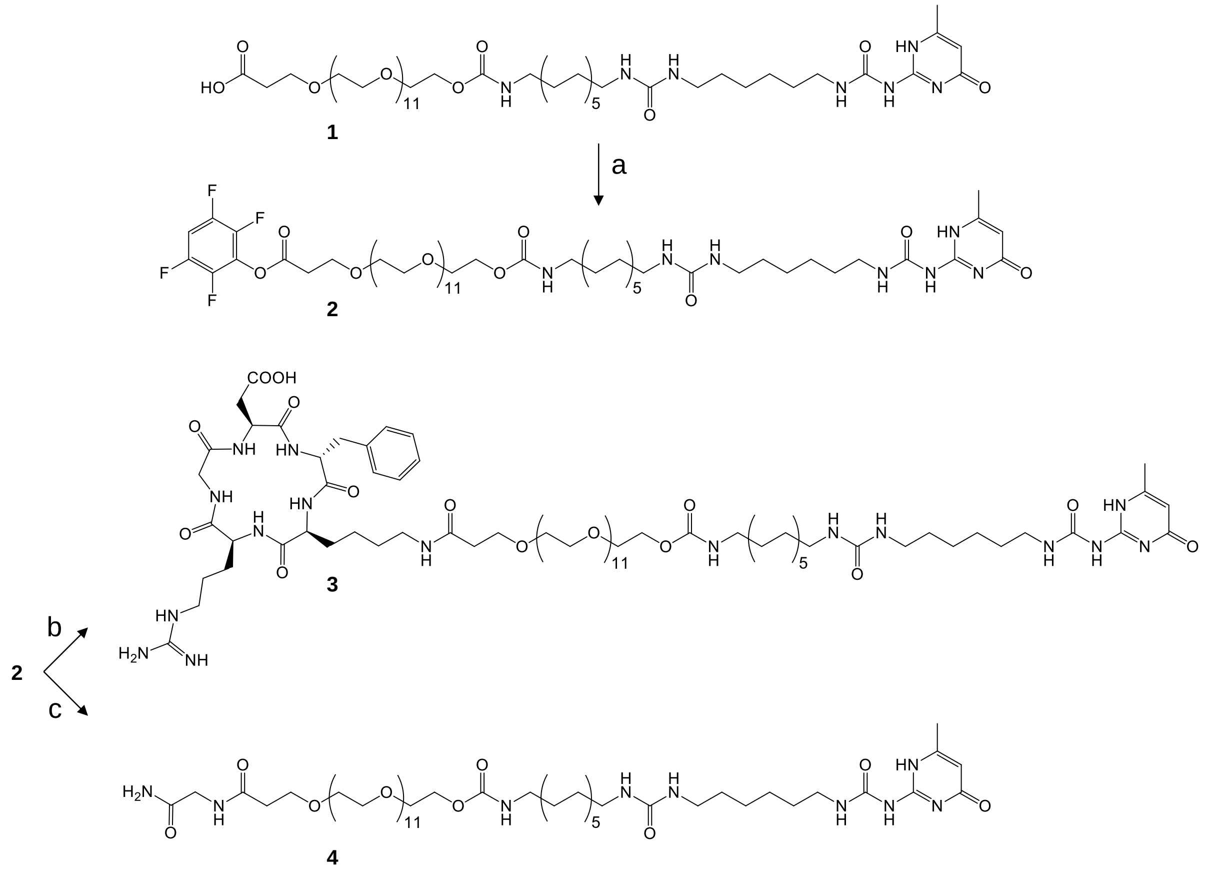

(**a**) 2,3,5,6-Tetrafluorophenol, pyridinium-*p*-toluenesulfonate, 1-ethyl-3-(3-dimethylaminopropyl)carbodiimide hydrochloride, $CHCl_3$, r.t., 2 hr, 100%. (**b**) c(RGDfK), *N*,*N*-diisopropylethylamine, DMF, r.t., 1 hr, 70%. (**c**) glycinamide hydrochloride, *N*,*N*-diisopropylethylamine, $CHCl_3$ / DMF 1:2, r.t., 1 hr, 93%.

2,3,5,6-Tetrafluorophenyl 1-((6-methyl-4-oxo-1,4-dihydropyrimidin-2-yl)amino)-1,10,25-trioxo-26,29,32,35,38,41,44,47,50,53,56,59,62-tridecaoxa-2,9,11,24-tetraazapentahexacontan-65-oate (**2**)

1-((6-Methyl-4-oxo-1,4-dihydropyrimidin-2-yl)amino)-1,10,25-trioxo-26,29,32,35,38,41,44,47,50,53,56,59,62-tridecaoxa-2,9,11,24-tetraazapentahexacontan-65-oic acid (**1**)[1] (41 mg, 36 µmol), 2,3,5,6-tetrafluorophenol (12 mg, 70 µmol, 2 eq) and pyridinium-*p*-toluenesulfonate (1.0 mg, 4 µmol, 0.1 eq) were dissolved in $CHCl_3$ (500 µL). 1-Ethyl-3-(3-dimethylaminopropyl)carbodiimide hydrochloride (14 mg, 72 µmol, 2 eq) was added and the solution was stirred at room temperature for 2 hr. $CHCl_3$ (50 mL) was added and the solution was washed with water (3 × 20 mL). The combined organic layers were dried using Na_2SO_4 and filtrated. Evaporation of the solvent *in vacuo* yielded pure **2** (46 mg, 36 µmol, 100%) as a

colorless solid. ^{1}H-NMR ($CDCl_3$): δ = 13.16 (br s, 1H, N*H*, UPy), 11.82 (br s, 1H, N*H*, UPy), 10.07 (br s, 1H, N*H*, UPy), 7.01 (m, 1H, Ar*H*), 5.83 (s, 1H, C=C*H*, UPy), 4.91 (br t, 1H, N*H*), 4.83 (br t, 1H, N*H*), 4.63 (br t, 1H, N*H*), 4.20 (t, 2H, NHC(O)OCH_2), 3.89 (t, 2H, NHC(O)OCH$_2$CH_2), 3.71–3.56 (m, 46H, OCH_2), 3.24 (q, 2H, CH_2NHC(O)O), 3.15 (m, 6H, CH_2NHC(O)NH), 2.96 (t, 2H, CH_2C(O)O), 2.24 (s, 3H, CH_3), 1.64–1.20 (m, 28H, CH$_2$CH_2CH$_2$). ESI-MS: *m/z* Calc. for $C_{59}H_{99}F_4N_7O_{19}$ 1285.69; Obs. $[M+2H]^{2+}$ 643.92, $[M+H]^{+}$ 1286.42, $[M+Na]^{+}$ 1308.42.

UPy-cRGD (**3**): 2-((2S,5R,8S,11S)-5-Benzyl-11-(3-guanidinopropyl)-8-(1-((6-methyl-4-oxo-1,4-dihydropyrimidin-2-yl)amino)-1,10,25,65-tetraoxo-26,29,32,35,38,41,44,47,50,53,56,59,62-tridecaoxa-2,9,11,24,66-pentaazaheptacontan-70-yl)-3,6,9,12,15-pentaoxo-1,4,7,10,13-pentaazacyclopentadecan-2-yl)acetic acid

A solution of **2** (113 mg, 88 µmol) in DMF (1.4 mL) was added to a stirring solution of c(RGDfK) (double TFA-salt, 103 mg, 0.12 mmol, 1.4 eq) and *N*,*N*-diisopropylethylamine (93 µL, 0.53 mmol, 6 eq) in DMF (0.4 mL). After stirring at room temperature for 1 hr the reaction mixture was precipitated in diethyl ether (60 mL). Centrifugation (5 min at 4000 rpm) was followed by decantation and the solid was washed with ether (10 mL). The centrifugation procedure was repeated and the resulting solid was dried *in vacuo* for 1 hr. The compound was purified with preparative RP-HPLC using a gradient of 33 to 36% MeCN in H_2O (both containing 0.1 v/v% formic acid). Lyophilization yielded pure **3** (106 mg, 61 µmol, 70%) as a white fluffy solid. ESI-MS: *m/z* Calc. for $C_{80}H_{138}N_{16}O_{25}$ 1723.00; Obs. $[M+3H]^{3+}$ 575.42, $[M+2H]^{2+}$ 862.67, $[M+H]^{+}$ 1723.42.

UPy-G (**4**): 42-Amino-39,42-dioxo-3,6,9,12,15,18,21,24,27,30,33,36-dodecaoxa-40-azadotetracontyl (12-(3-(6-(3-(6-methyl-4-oxo-1,4-dihydropyrimidin-2-yl)ureido)hexyl)ureido)dodecyl)carbamate

A solution of **2** (618 mg, 0.48 mmol) in $CHCl_3$ / DMF 1:2 (5 mL) was added to a stirring solution of glycinamide hydrochloride (66 mg, 0.59 mmol, 1.2 eq) and *N*,*N*-diisopropylethylamine (0.50 mL, 2.8 mmol, 6 eq) in DMF (0.5 mL). After stirring at room temperature for 1 hr the solvent was removed *in vacuo* (oil pump, 45 °C). The resulting solid was dissolved in $CHCl_3$ / MeOH 9:1 (250 mL) and washed with H_2O / brine 1:1 (2 × 100 mL). After drying with Na_2SO_4 and filtration, the filtrate was evaporated to dryness and the resulting solid was flushed with $CHCl_3$ (2 × 20 mL). The solid was re-dissolved in $CHCl_3$ / MeOH 3:1 (4 mL) and precipitated in diethyl ether (35 mL). Centrifugation (5 min at 4000 rpm) was followed by decantation and the solid was washed with ether (20 mL).

Centrifugation and decantation were repeated and the solid was re-dissolved in $CHCl_3$ / MeOH 3:1 (4 mL). The entire precipitation-centrifugation procedure was repeated once and the resulting solid was dried *in vacuo* for 16 h, yielding pure **4** (534 mg, 0.45 mmol, 93 %) as a white solid. ESI-MS: *m/z* Calc. for $C_{55}H_{103}N_9O_{19}$ 1193.74; Obs. $[M+2H]^{2+}$ 597.92, $[M+H]^{+}$ 1194.58, $[M+Na]^{+}$ 1216.58.

Supplementary Method 2: Synthesis of UPy-Cy5

Materials:

Reverse-phase high-performance liquid chromatography–mass spectrometry (RP-HPLC–MS) was performed on an LCQ Fleet ion trap mass spectrometer. Sulfo-Cy5-NH_2 was purchased from Lumiprobe (USA).

Synthesis:

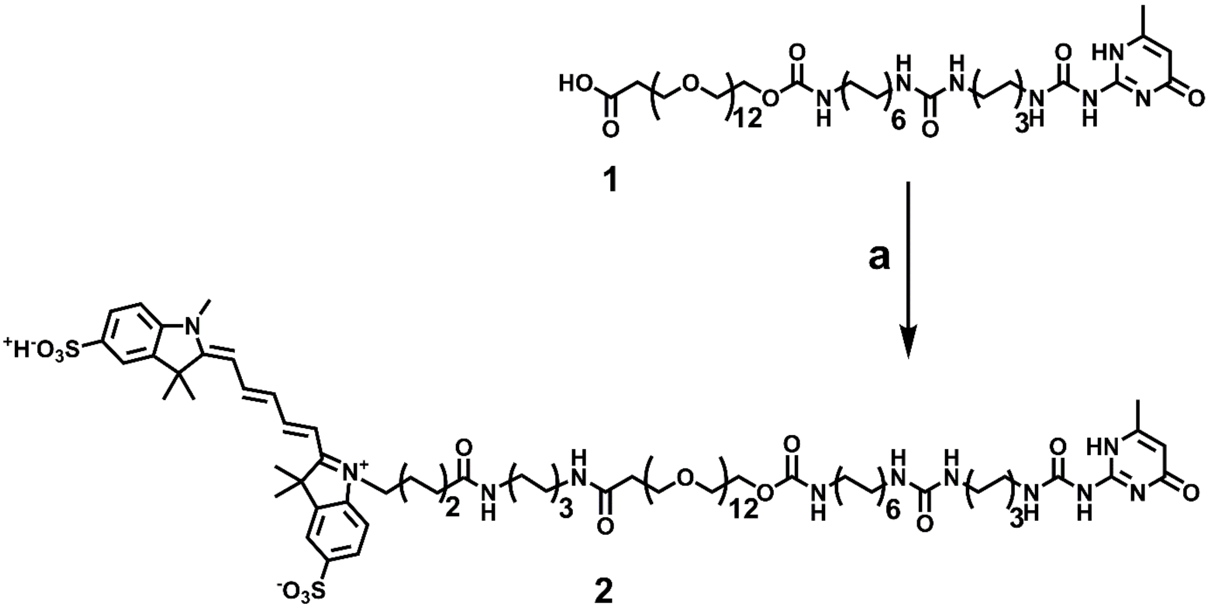

(**a**) 1-[Bis(dimethylamino)methylene]-1H-1,2,3-triazolo[4,5-b]pyridinium 3-oxid hexafluorophosphate, Hexafluorophosphate Azabenzotriazole Tetramethyl Uronium, N-N-diisopropylethylamine, Suflo-Cy5-NH_2, DMF, r.t., 1 hr, 45%.

1-[6-[(6-aminohexyl)amino]-6-oxohexyl]-2-[5-(1,3-dihydro-1,3,3-trimethyl-5-sulfo-2H-indol-2-ylidene)-1,3-pentadien-1-yl]-3,3-dimethyl-5-sulfo 1-((6-methyl-4-oxo-1,4-dihydropyrimidin-2-yl)amino)-1,10,25-trioxo-26,29,32,35,38,41,44,47,50,53,56,59,62-tridecaoxa-2,9,11,24-tetraazapentahexacontan-65-oate (**2**)

1-((6-Methyl-4-oxo-1,4-dihydropyrimidin-2-yl)amino)-1,10,25-trioxo-26,29,32,35,38,41,44,47,50,53,56,59,62-tridecaoxa-2,9,11,24-tetraazapentahexacontan-65-oic acid (**1**)[1] (2.36 mg, 20.8 µmol), 1-[Bis(dimethylamino)methylene]-1H-1,2,3-triazolo[4,5-b]pyridinium 3-oxid hexafluorophosphate, Hexafluorophosphate Azabenzotriazole Tetramethyl Uronium (1.58 mg, 41.6 µmol) was dissolved in DMF (2 mL). N,N-Diisopropylethylamine (2.15 mg, 16.6 µmol) was added and the solution was stirred at room temperature for 15 min. Sulfo-Cy5-NH_2 (2 mg, 27.0 µmol) dissolved in DMF (3 mL) was added to the solution and stirred for 1 hr in an argon environment. H_2O (containing 0.1 v/v% formic acid, 20 mL) was added to the solution and centrifuged (4 min at 3000 rpm) followed by decantation. Ultrapure water was added (20 mL) and the product was lyophilized. The compound was purified with preparative RP-HPLC using a gradient of 40% ACN in H_2O (both containing 0.1 v/v% trifluoroacetic acid). Lyophilization yielded pure **2** (1.75 mg, 9.4 µmol, 45%) as a blue solid. ESI-MS: *m/z* Calc. for $C_{91}H_{149}N_{11}O_{25}S_2$ 1861.37; Obs. $[M+3H]^{3+}$ 621.33, $[M+2H]^{2+}$ 931.17, $[M+H]^{+}$ 1861.75. UPy-Cy3 additives were synthesis in a similar manner.

Supplementary Method 3: Förster resonance energy transfer (FRET) measurements

Instrument:

A Varian Fluorescence spectrophotometer was used to record the fluorescence emission scans with an excitation wavelength of 540 nm. For these measurements, the emission was recorded from 550 to 750 nm, the emission and excitation slits were set at 5 nm, and a scan rate of 600 nm/min was employed. Additionally, a voltage of 1000 V was set for the photomultiplier tube (PMT) detector. The measurements were performed at 20 °C.

Sample preparation:

Stock solutions of 25 µM UPy-G or UPy-PEG-UPy building blocks in PBS were prepared by vortexing and stirring for 90 min at 75 °C until a clear solution was obtained. Subsequently, 250 µM stock solutions of the UPy-functionalized fluorescent dyes (UPy-Cy3 and UPy-Cy5; both sulfonated) were prepared in PBS. Subsequently, 5 µL of one of the UPy-dye solutions was added to 495 µL of either the M- or B-type building block solutions, reaching final concentrations of 2.5 µM of UPy-dye and 24.75 µM of M- or B-type building blocks. The dye-building block mixture was then vortexed and annealed at 80 °C for 10 minutes. Subsequently, the mixture was kept at room temperature overnight to allow for the incorporation of the dyes into the supramolecular stacks overnight. On the next day,

immediately prior to each FRET measurement, two solutions of the same building block type (with either dye) were mixed at a 1:1 volumetric ratio.

Fluorescence measurements:

Emission scans were caried out using a 24.75 µM concentration of supramolecular building blocks supplemented with UPy-Cy3 (1.25 µM) and/or UPy-Cy5 (1.25 µM) additives. These measurements were caried out at an excitation wavelength of 540 nm, and using emission scans between 550 and 750 nm.

Upon mixing two dispersions of supramolecular building blocks with different dyes (UPy-Cy3 or UPy-Cy5), emission scans were recorded every minute in the first 10 minutes and subsequently every 10 or 15 minutes. The ratiometric FRET ratio was calculated according to the following equation,

$$\text{FRET ratio}=\frac{A}{(A+B)}$$

where A and B are the emission signals at 670 nm (UPy-Cy5 acceptor) and 565 nm (UPy-Cy3 donor) wavelength, respectively.

Supplementary Method 4: *In vitro* degradation and release behavior

Hydrogels (100 µL) containing 100 µM of UPy-Cy5 or Cy5 were formed at the bottom of 2 mL glass vials through pH-induced gelation. 500 µL of PBS were added to each vial, and the vials were incubated at 37 °C for up to 7 days. At each time point, the solution was collected using a pipette, the extra water at the surface of hydrogels was removed using tissue wipers, the weight of the hydrogels was recorded using a microbalance to determine their weight change, and 500 µL of fresh PBS were added into each vial. After the final time point, the hydrogels were dried in a vacuum oven at 60 °C overnight, after which their dry mass was measured using a microbalance to determine their erosion. The concentration of released UPy-Cy5 or Cy5 molecules in solutions was determined by measuring the fluorescence intensity using a Tecan Safire^2 microplate reader (646 nm excitation, 662 nm emission). The amount of released dye (UPy-Cy5 or Cy5) was calculated as a percentage of the initial dye content incorporated in the hydrogels.

Supplementary Method 5: Organoid formation and culture

Human hepatocyte organoid culture: Human hepatocyte organoids isolated from human fetal tissue were cultured on Matrigel based on previous work by Clevers et al.[2] Briefly, 20,000-

50,000 cells were suspended in Matrigel, and 10 droplets of 10 µL were plated in a 6-well plate. After Matrigel was solidified (>20 min), 1 mL of human hepatocyte medium was added per well. Human hepatocyte medium consisted of Advanced DMEM/F12 (Thermo Fisher Scientific, with HEPES, GlutaMax and penicillin-streptomycin) and 15% RSPO1 conditioned medium (home-made), B27 (minus vitamin A), 50 ng mL^{-1} EGF (Peprotech), 1.25 mM N-acetylcysteine (Sigma), 10 nM gastrin (Sigma), 3 mM CHIR99021 (Sigma), 50 ng mL^{-1} HGF (Peprotech), 100 ng mL^{-1} FGF7 (Peprotech), 100 ng mL^{-1} FGF10 (Peprotech), 2 mM A83-01 (Tocris), 10 mM Nicotinamide (Sigma-Aldrich), 10 mM Rho Inhibitor g-27632 (Calbiochem) and 20 ng mL^{-1} TGFa. During the culture period, the medium was refreshed every 2-3 days, and the organoids were passaged with a split ratio of 1:3 every 14 days.

Organoid encapsulation in hydrogels: Pre-cultured human hepatocyte organoids in Matrigel were split in a 1:3 ratio and encapsulated in either supramolecular hydrogels or Matrigel. Organoids were resuspended in growth medium (60 µL) and mixed with the B dispersion (120 µl, 0.75 wt%) in an Eppendorf tube. The organoid encapsulation in supramolecular hydrogels was then carried out through the mixing-induced gelation method. To this end, an M dispersion was mixed with the B dispersion (+organoids) inside an Eppendorf tube through gentle pipetting for ~1 min. Thereafter, 50 µL of the mixture was pipetted in 3 droplets per well in a 12-well plate. The Matrigel control was prepared separately and plated as 50 µl in 3 droplets in a 12-well plate. All conditions were plated in triplicate. After 30 minutes of incubation at 37 °C and 5% CO_2 atmosphere, 1 mL of hepatocyte medium was slowly added to each well. The medium was refreshed at Day 5, and brightfield images were recorded at 1, 5 and 7 days. An ATP assay was performed on Day 7 using a CellTiter-Glo® 3D Cell Viability Assay (Promega®). The data was normalized by the number of organoids present. The change in surface area was determined by measuring the area of 30 organoids in ImageJ (Figure S15, Supporting Information) on both Day 1 and Day 7 of the same droplet in the same well, and calculating the fold increase (FI) in surface area using the following equation: $FI = ([Area_{Day\ 7}]-[Area_{Day\ 1}])/[Area_{Day\ 1}]$.

Supplementary References:

[1] I. de Feijter, O. J. G. M. Goor, S. I. S. Hendrikse, M. Comellas-Aragonès, S. H. M. Söntjens, S. Zaccaria, P. P. K. H. Fransen, J. W. Peeters, L.-G. Milroy, P. Y. W. Dankers, *Synlett* **2015**, *26*, 2707.

[2] H. Hu, H. Gehart, B. Artegiani, C. LÖpez-Iglesias, F. Dekkers, O. Basak, J. van Es, S. M. Chuva de Sousa Lopes, H. Begthel, J. Korving, M. van den Born, C. Zou, C. Quirk, L. Chiriboga, C. M. Rice, S. Ma, A. Rios, P. J. Peters, Y. P. de Jong, H. Clevers, *Cell* **2018**, *175*, 1591.
